# The oxidative potential of differently charged silver and gold nanoparticles on three human lung epithelial cell types

**DOI:** 10.1186/s12951-014-0062-4

**Published:** 2015-01-16

**Authors:** Paul Schlinkert, Eudald Casals, Matthew Boyles, Ulrike Tischler, Eva Hornig, Ngoc Tran, Jiayuan Zhao, Martin Himly, Michael Riediker, Gertie Janneke Oostingh, Victor Puntes, Albert Duschl

**Affiliations:** Department of Molecular Biology, Paris Lodron-University of Salzburg, Hellbrunnerstr. 34, A-5020 Salzburg, Austria; Institute Catalá de Nanotecnologia, Barcelona, Spain; Institute for Work and Health, Lausanne, Switzerland; Institue for Occupational Medicine (IOM) Singapore, Downtown Core, Singapore; Biomedical Sciences, Salzburg University of Applied Sciences, Puch, Salzburg Austria

**Keywords:** Human lung epithelial cells, Nanoparticles, Cytotoxicity, ROS production, Surface charge

## Abstract

**Background:**

Nanoparticle (NPs) functionalization has been shown to affect their cellular toxicity. To study this, differently functionalized silver (Ag) and gold (Au) NPs were synthesised, characterised and tested using lung epithelial cell systems.

**Methods:**

Monodispersed Ag and Au NPs with a size range of 7 to 10 nm were coated with either sodium citrate or chitosan resulting in surface charges from −50 mV to +70 mV. NP-induced cytotoxicity and oxidative stress were determined using A549 cells, BEAS-2B cells and primary lung epithelial cells (NHBE cells). TEER measurements and immunofluorescence staining of tight junctions were performed to test the growth characteristics of the cells. Cytotoxicity was measured by means of the CellTiter-Blue ® and the lactate dehydrogenase assay and cellular and cell-free reactive oxygen species (ROS) production was measured using the DCFH-DA assay.

**Results:**

Different growth characteristics were shown in the three cell types used. A549 cells grew into a confluent mono-layer, BEAS-2B cells grew into a multilayer and NHBE cells did not form a confluent layer. A549 cells were least susceptible towards NPs, irrespective of the NP functionalization. Cytotoxicity in BEAS-2B cells increased when exposed to high positive charged (+65-75 mV) Au NPs. The greatest cytotoxicity was observed in NHBE cells, where both Ag and Au NPs with a charge above +40 mV induced cytotoxicity. ROS production was most prominent in A549 cells where Au NPs (+65-75 mV) induced the highest amount of ROS. In addition, cell-free ROS measurements showed a significant increase in ROS production with an increase in chitosan coating.

**Conclusions:**

Chitosan functionalization of NPs, with resultant high surface charges plays an important role in NP-toxicity. Au NPs, which have been shown to be inert and often non-cytotoxic, can become toxic upon coating with certain charged molecules. Notably, these effects are dependent on the core material of the particle, the cell type used for testing and the growth characteristics of these cell culture model systems.

**Electronic supplementary material:**

The online version of this article (doi:10.1186/s12951-014-0062-4) contains supplementary material, which is available to authorized users.

## Background

Various characteristics of nanoparticles (NPs) can influence their toxicity, such as size [1], shape [2] or surface coating [3]. In the present study, silver (Ag) and gold (Au) NPs of similar size were synthesised with different coatings to provide 4 classes of surface charge ranging from −50 mV to +70 mV. One class of negatively charged NPs, coated with sodium citrate, (Ag/Au-SC) and three classes (low, medium, high) of positively charged NPs, coated with chitosan, (Ag/Au-CHIT-L/M/H) were synthesised. These metal NPs were chosen, as they are in widespread use, Au NPs are particularly used in medical applications [4]. In contrast, Ag NPs were reported to have anti-microbial properties [5]. Furthermore, Ag NPs have been described in many studies to be cytotoxic for human cells [6-9], whereas Au NPs were mainly found to be inert and only few studies report cytotoxicity of Au NPs [10-12]. The surface coating used to achieve the positive charge is also of special interest, as chitosan-coated NPs are increasingly used in the field of nanobiotechnology. These type of functionalized NPs are very promising drug delivery systems [13,14], due to their low toxicity, high stability and biocompatibility [15]. The positive surface charge of these NPs renders them more suitable for an intravenous injection, as it has been reported that positively charged NPs remain in the blood stream longer than negatively charged NPs [16], which is a common route for the administration of anticancer agents [15]. Furthermore, chitosan NPs are also suitable to be administered orally and this administration route has been used for the delivery of drugs [17] and genes [18]. Finally, the inhalation of NPs appears to be a promising method for the delivery of drugs to the lung [19,20]

In a number of studies metal NPs were reported to induce oxidative stress (OS) [21-24]. However, it remains unclear which properties of NPs contribute to the induction of OS. Oxidative stress is a direct result of an imbalance of the cell’s redox potential, where reactive oxygen species (ROS) are produced at a rate that the cell’s antioxidant mechanisms are unable to detoxify [24]. The formation of ROS has also been linked to inflammation and apoptosis. Even though OS can also be induced by the production of reactive nitrogen species [25], this study focuses on the production of ROS. A cellular response to NPs may be dependent on the proteins from their surrounding biofluids which quickly adsorb to the NPs surface [26], first forming a weakly bound soft corona, which can be replaced by a hard corona over time [27]. This process is influenced by both the NPs properties and the composition of the solution [28]. Therefore, this study has examined whether NP functionalization, presenting different surface charges, can influence the interaction of NPs with cell culture medium components and in turn influence ROS production. The potential for some NPs to produce ROS directly on their surface is well recognized [29,30], however, it is important to determine whether this occurs under conditions as they exist in cell culture, which was performed in this study.

In vitro cell exposures provide a vital tool to assess potential risks to humans as these techniques reduce the need for animal studies and provide the opportunity to use human cells; so despite being artificial, a good understanding of in vitro cell systems is necessary. Regarding accidental exposure, inhalation is the most likely route [31]. Deposition within the lung is either due to interception, impaction, sedimentation or diffusion of particles, which is dependent on the size of the particles [32]. Large micron sized particles mainly deposit in the nasopharyngeal region (5–30 μm) as a result of impaction and interception and are then subject to mucociliary clearance [33]. Sedimentation of NPs commonly only occurs with particles with a diameter above 0.5 μm, whereas the deposition of NPs within the lung is mostly due to diffusion [32]. In contrast to larger particles, NPs have been shown to travel deeper into the lung [32]. Several studies report deposition of NPs within the tracheobronchial region [32,34,35], but also within the deepest region of the lung, the alveolar region [36,37]. Once deposited in the alveolar region, clearance of small NPs has been proven difficult [38] and exposure to the NPs is thereby prolonged [39]. The prolonged exposure will allow the NPs to directly interact with the epithelial layer of the alveolar region, which can in turn lead to translocation of NPs into the blood stream and the subsequent deposition in other organs [40]. However, it has also been shown that insoluble NPs can remain in the lung indefinitely [41], thereby significantly increasing the risk of adverse effects.

The NPs used in this study are in a size range where they can deposit in the tracheobronchial as well as within in the alveolar region [35]. We therefore chose three different human lung epithelial cell types to assess the effects of NP exposure to the human lung for this study, which represent both the tracheobronchial and alveolar regions.

Two stable cell lines were used: the human alveolar adenocarcinoma cell line (A549) [42] and the human bronchial epithelial cell line (BEAS-2B) [43]. In addition, primary human bronchial epithelial cells (NHBE) [44], derived from healthy donors, were used since they represent the *in vivo* system more closely than the cell lines. These cell types are derived from different parts of the lung and have different properties. A549 cells are of interest since they originate from type II alveolar epithelial cells and not from bronchia, while the other two cell types do [45]. Even though alveolar epithelial cells are not covered by a mucosal layer, they produce a surfactant layer *in vivo*, which provides additional protection [46]. A549 cells are an important, well-established cell line and frequently used as a model in the assessment of NPs induced lung cytotoxicity, which is illustrated by the high number of publications that mention A549 cells and nanoparticles. In addition, A549 cells rapidly grow under submerged cell culture conditions that allows them to be used in high throughput screenings. The advantage of NHBE cells is that they are primary cells derived from healthy lung tissue, while BEAS-2B cells have the advantage of readily forming a tight epithelium. All three cell types require different culture media, bringing in further deviations to the *in vivo* situation. In light of their respective benefits and drawbacks it is likely that no single cell type will emerge as universal model in nanosafety research. The three cell types were used since they have all been used for studies on the nanosafety of inhaled NPs [47,48]. A comparison between them is especially useful as NPs that enter the respiratory system may deposit throughout the airways and lung sections, therefore contact with different types of lung cells is relevant.

## Results

### Cell development

**Figure 1:**
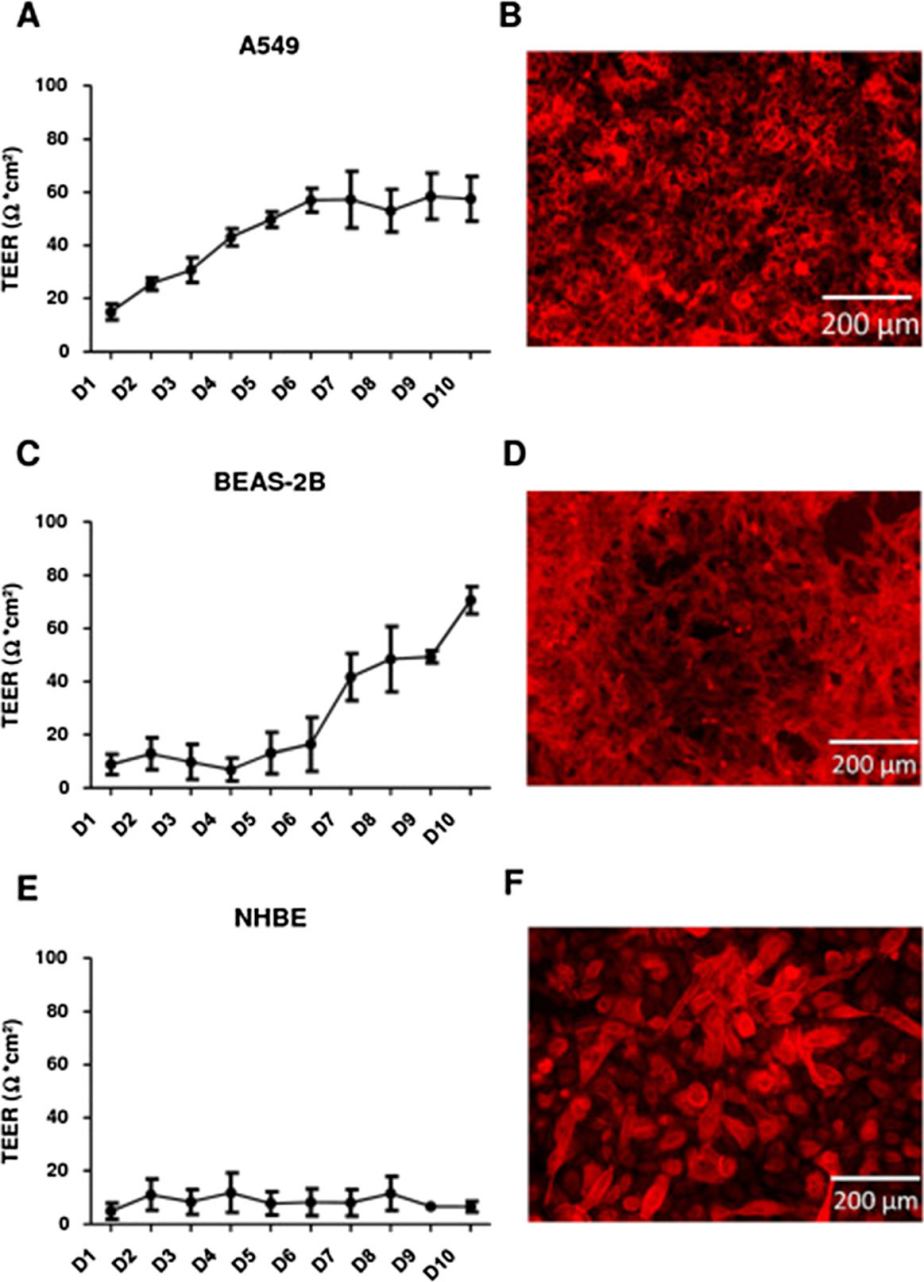
**Development of the epithelial layer in (A-B) A549 cells, (C-D) BEAS-2B cells and (E-F) NHBE cells.** TEER measurements **(A, C and** **E)** show the means ± SD of a minimum of 3 experiments. Staining of tight junction proteins: Claudin-1 staining **(B)** in A549 cells at day 4, **(D)** in BEAS-2B cells at day 7 and **(F)** in NHBE cells at day 7. All pictures were taken with a 10x magnification.

Understanding the growth characteristics of the cell types used in this study is important in order to fully comprehend the observed responses to NPs insult. Epithelial cells grow in monolayers *in vivo* and therefore a tightly formed and well-functioning monolayer is preferred for *in vitro* experiments to increase the similarity to lung epithelia *in vivo*. TEER measurements and fluorescence microscopy using immunodetection of claudin-1 as a marker for tight junction proteins were performed to follow the formation of a tight cell layer. Figure 1a shows that A549 cells, when grown from a seeding density of 1x10^5^ cells/ml, formed an intact monolayer after four days and remained stable for several days as a monocellular layer. The first successful staining of tight junction proteins was also achieved at day 4 (Figure 1b). In contrast, BEAS-2B cells plated at the same cell density did not form a confluent monolayer until day 7 (Figure 1c). A different growth pattern was observed for BEAS-2B cells, which were shown to grow on top of each other and formed multilayers which also contained functional tight junctions (Figure 1d). This resulted in a tight epithelial cell layer, but the multiple cell layer phenotype does not correspond to *in vivo* situations. NHBE cells did not grow into a monolayer under our culture conditions, as maximum TEER values of only 12 Ω*cm^2^ were determined (Figure 1e), while values of 67 Ω*cm^2^ and 75 Ω*cm^2^ were determined for A549 and BEAS-2B cells respectively (Figure 1a, c). NHBE cells did, however, synthesise the proteins necessary for the formation of tight junctions. Yet, the proteins were only found in the centre of the cell and failed to move to the cell membrane where they would be needed for the formation of tight junctions (Figure 1f). This difference between cell lines of similar origin is also evident in other cell types as well and should be carefully monitored before performing a study [49]. All three cell types used here represent certain aspects of epithelia in the lung, but clearly display different properties.

### Cytotoxicity

#### Effects of functionalized NPs on the cell membrane integrity

**Figure 2:**
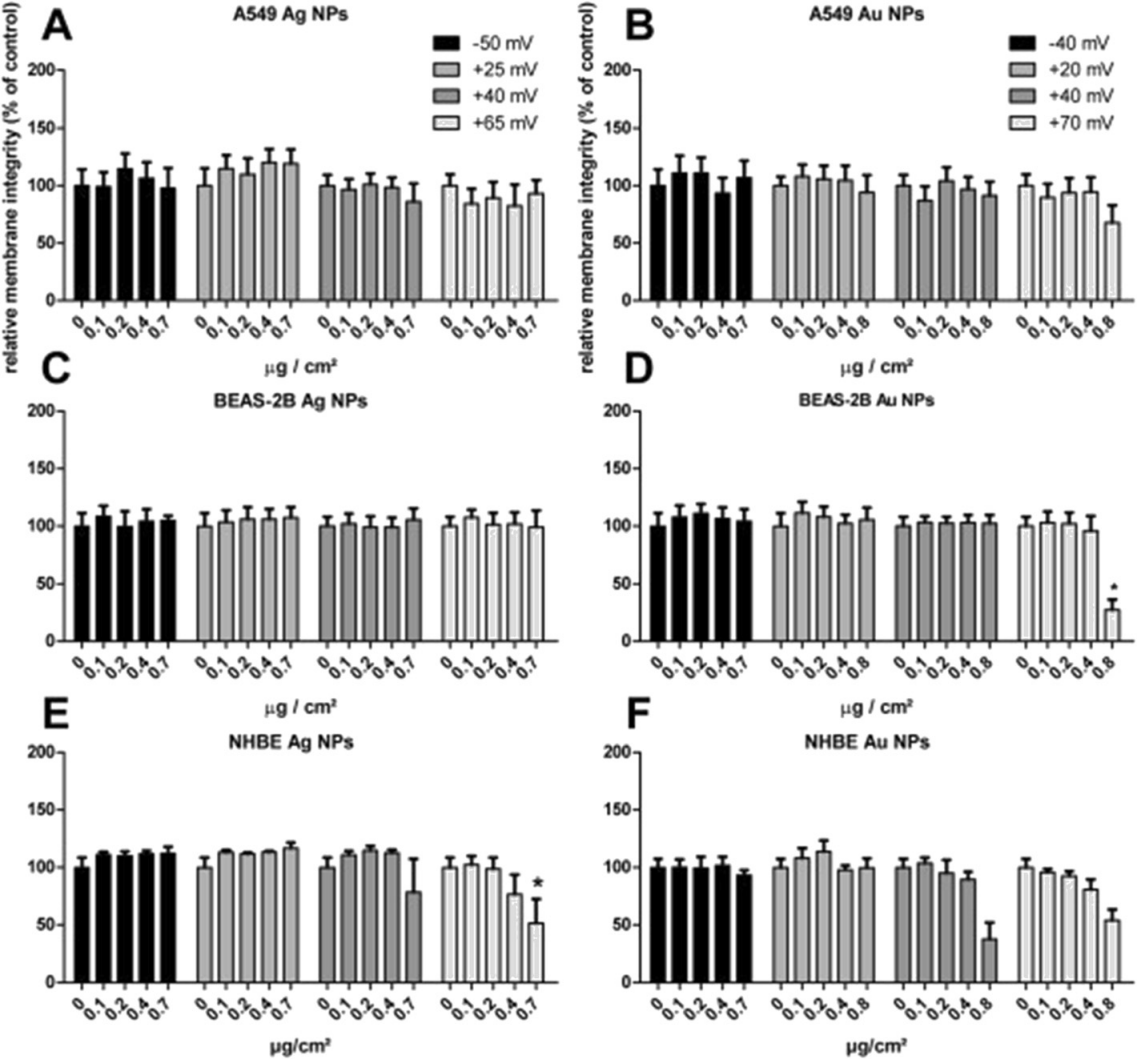
**Cell membrane integrity, as measured by an increase in LDH-release, following a 24 h exposure of the different cell lines to Ag and Au NPs.** An increase in LDH-release is indicated by a decrease in the membrane integrity. A549 cells **(A, B**; means ± SEM of n = 6), BEAS-2B **(C, D**; means ± SEM of n = 3) and NHBE cells **(E, F**; means ± SEM of n = 3). P-value * < 0.05. Cells treated with medium only were used as negative control (=100%).

When A549 cells were exposed to increasing concentrations of differently functionalized Ag or Au NPs for 24 hours, no increase in LDH release was observed (Figure 2a). Only exposure to the Au NPs with the highest amount of chitosan (Au-CHIT-H) induced a small increase in LDH release, which was statistically not significant (Figure 2b). The same findings were observed at exposure periods of 4 and 48 hours (Additional files 1 and 2).

Similar results were observed upon exposure of BEAS-2B cells for 24 hours. Here, only the highest charged Au NPs (Au-CHIT-H) at a high concentration resulted in membrane impairment (Figure 1c, d). This increase in LDH release was also observed after 4 and 48 hour exposures (Additional files 1 and 2).

In contrast, NHBE cells were more susceptible towards both Ag and Au NPs. An increase in LDH release was observed at high concentrations (0.4 and 0.8 μg/cm^2^) of chitosan-coated Ag NPs (Ag-CHIT-M/H). The two Au NP-preparations coated with the largest amount of chitosan (Au-CHIT-M/H) were shown to increase LDH release, without reaching statistical significance. A shorter exposure of 4 hours did not induce any membrane leakage (Additional file 1), yet a longer period of 48 hours showed the same trends as found after 24 hours (Additional file 2).

In addition, the solvents, sodium citrate and chitosan, were also tested with all cells and for each time point and no effects on membrane integrity or cell viability were observed. Furthermore, no interference of the NPs with the assay, such as binding of the end product, was observed (Additional file 3).

#### Effects of functionalized NPs on cell viability

**Figure 3:**
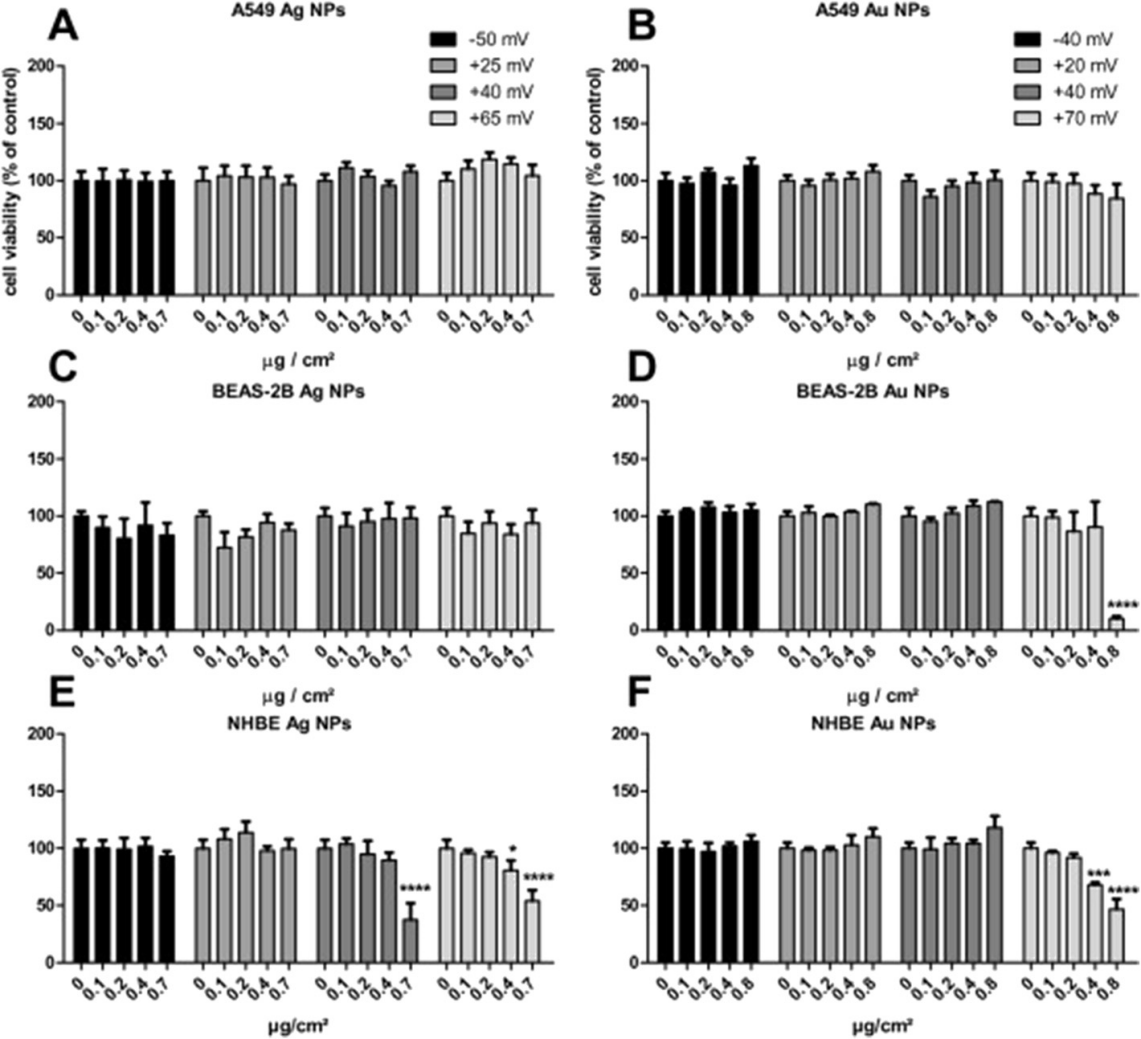
**Cell viability following a 24 h exposure to functionalized Ag and Au NPs of A549 cells (A, B; means ± SEM of n = 6), BEAS-2B cells (C, D; means ± SEM of n = 3) and NHBE cells (E, F; means ± SEM of n = 3).** P-values * < 0.05, *** < 0.005, **** < 0.001. Cells treated with medium only were used as control (100%).

During a 24-hour exposure period, neither Ag nor Au NPs were shown to reduce the viability of A549 cells (Figure 3a,b). The same was observed after 4 and 48 hour exposures (Additional files 4 and 5).

Differently functionalized Ag NPs did not induce a significant decrease in cell viability in BEAS-2B cells after 24 hours (Figure 3c). Most of the chitosan-coated Au NPs showed no effect, however, a concentration of 0.8 μg/cm^2^ of Au NPs with the highest amount of chitosan (Au-CHIT-H) induced a significant decrease in cell viability (Figure 3d). The same trends were found at other time points (Additional files 4 and 5). These data are in agreement with those found for the LDH assay (Additional files 1 and 2).

The highest responses to functionalized Ag and Au NPs were observed for NHBE cells. Here, a significant decrease in cell viability was observed when NHBE cells were exposed to high concentrations of chitosan-coated Ag (Ag-CHIT-M/H) and Au NPs (Au-CHIT-H) for 24 hours (Figure 3e, f). These findings are in line with those of the LDH assay. When the cells were exposed for only 4 hours, no decrease in cell viability was observed (Additional file 4). In contrast, increasing the exposure period to 48 hours induced a decrease in viability by the same NPs as after 24 hours (Additional file 5).

In contrast to the LDH assay, where no interference was found, there is a small interference of the light emitted during the endpoint measurement of the CTB assay, by both Ag and Au NPs coated with a high amount of chitosan (Additional file 6). There is an increase in the fluorescence signal when these particles are present during the measurement, which may be incorrectly interpreted as an increase in cell viability. This effect may have caused a small underestimation of the NP-induced reduction in cellular viability.

### Oxidative stress

#### Induction of intracellular ROS production by functionalized NPs

To analyse the oxidative stress induced by NP exposure, shorter exposure periods compared to those of the cytotoxicity assays were chosen, since cell-mediated ROS production by NPs is a rapid process, which might be lost at later time points.

**Figure 4:**
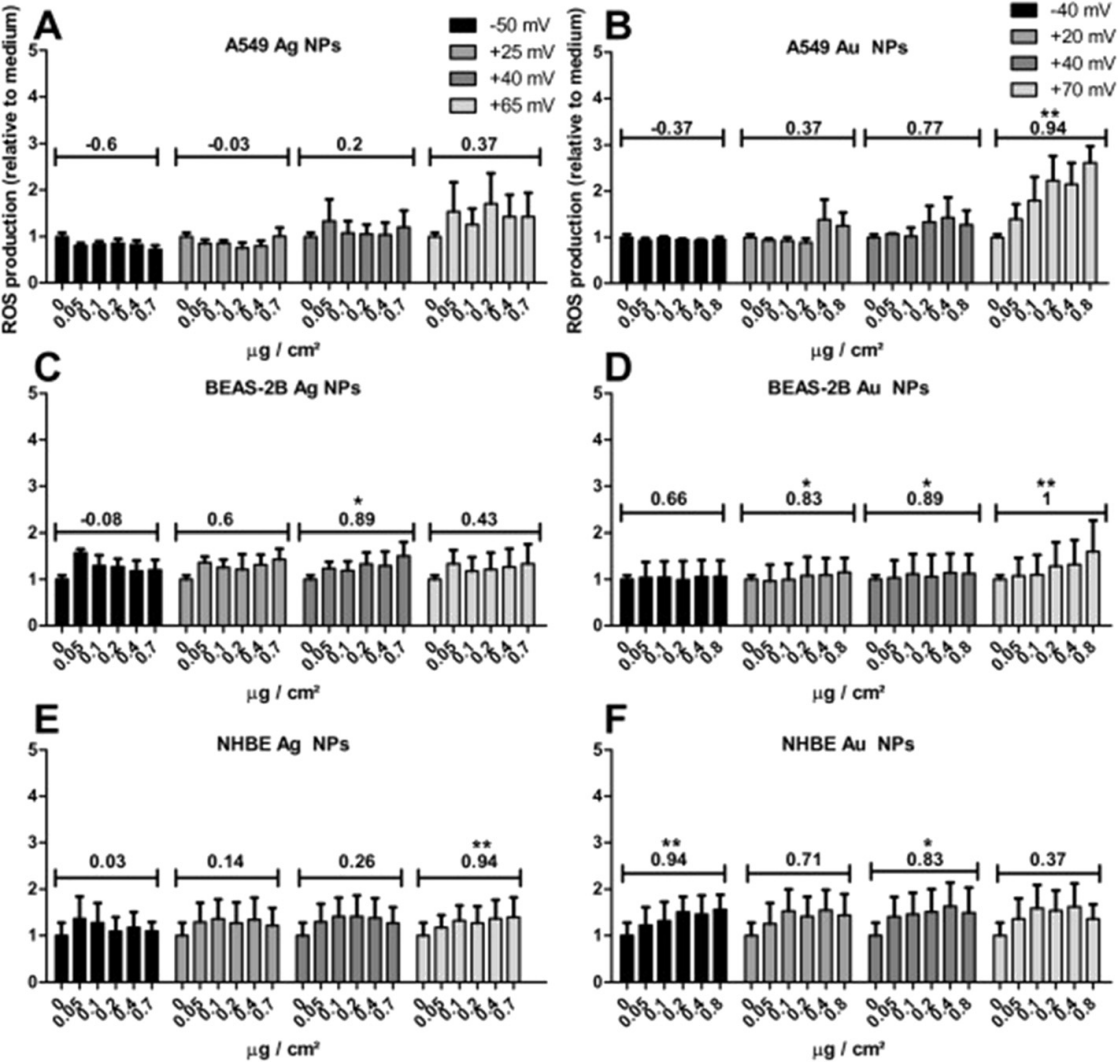
**ROS production measured using the DCFH-DA assay following a 4 h exposure of A549 cells (A, B, means ± SEM of n = 3), BEAS-2B cells (C, D, means ± SEM of n = 3) and NHBE cells (E, F means ± SEM of n = 4) to charged Ag and Au NPs.** Spearman’s rank coefficients were calculated for each NP to assess possible charge dependent increases in ROS production. In addition, the coefficients of the highest concentrations of each NP surface charge were calculated to determine if ROS production was charge dependent. P-values * < 0.05, ** < 0.01.

Exposing A549 cells to chitosan coated Ag NPs (Ag-CHIT-L/M/H) for 4 hours induced low levels of ROS production, with the highest levels observed for the highest amount of chitosan (Ag-CHIT-H) (Figure 4a). In contrast, a significant concentration-dependent increase in ROS production, as determined by Spearman’s rank correlation, could be observed following the exposure Au NPs with the highest amount of chitosan on their surface (Au-CHIT-H), whereas exposure to Au NPs with less amount of chitosan (Au-CHIT-L/M) only resulted in a small increase in ROS production (Figure 4b). The same observations were made when cells were exposed for 1 hour (Additional file 7).

Interestingly, both Ag and Au NPs induced some degree of ROS production after a 4 hour exposure (Figure 4c,d) in BEAS-2B cells, however, this was less prominent when compared to a 1 hour exposure (Additional file 7). Furthermore, ROS production induced by Au NPs with a large amount of chitosan (Au-CHIT-H) was not as high as that seen in A549 cells, yet a significant concentration dependency was still determined by Spearman’s rank correlation.

NPs induced the least ROS production in NHBE cells. Only small amounts of ROS were induced by Ag NPs after 4 hours (Figure 4e) and only slightly higher in response to Au NPs. However, a NPs concentration dependency was observed following the exposure to sodium citrate-coated Au NPs (Au-SC) (Figure 4f). Similar amounts of ROS were produced after 1 hour, yet here a concentration dependency could be observed for all of the NPs studied (Additional file 7).

Additionally, Spearman’s rank coefficients were determined to assess if NP-induced ROS production was dependent on functionalization. The assessment can be viewed in Tables 1 and 2. The ROS production induced by both Ag and Au NPs in A549 cells appeared to be functionalization-dependent, as statistically significant Spearman’s rank coefficients were found with a change from negatively to positively charged surface coatings and with further increases in positive charge, evident in all NPs concentrations. This is in contrast to BEAS-2B, where no correlation between Ag NPs induced ROS production was found and only in response to higher concentrations of Au NPs. In NHBE cells, where ROS production could only be correlated to Ag NPs functionalization at higher NPs concentrations, while Au NPs did induce ROS production in a functionalization-dependent manner at all concentrations except the highest.

**Table 1:** **Overview of Spearman’s rank coefficients to assess charge-dependent increase in ROS production following a 4 hour exposure to differently charged Ag NPs**

**NPs (μg/cm** ^**2**^ **)**	**A549**	**BEAS-2B**	**NHBE**
0	0	0	0
0.05	1^**^	−0.20	−0.2
0.1	1^**^	−0.33	1
0.2	0.87^*^	0.07	0.60
0.4	0.87^*^	0.47	0.87^*^
0.8	1^**^	0.60	1^*^

P-values * < 0.05, ** < 0.01.

**Table 2:** **Overview of Spearman’s rank coefficients to assess charge-dependent increase in ROS production following a 4 hour exposure to differently charged Au NPs**

**NPs (μg/cm** ^**2**^ **)**	**A549**	**BEAS-2B**	**NHBE**
0	0	0	0
0.05	1^**^	0.60	0.87^*^
0.1	0.87^*^	0.87^*^	0.87^*^
0.2	0.87^*^	0.60	0.87^´*^
0.4	1^**^	1^**^	0.87^*^
0.8	1^**^	0.87^*^	−0.2

P-values * < 0.05, ** < 0.01.

#### ROS production in a cell-free system

**Figure 5:**
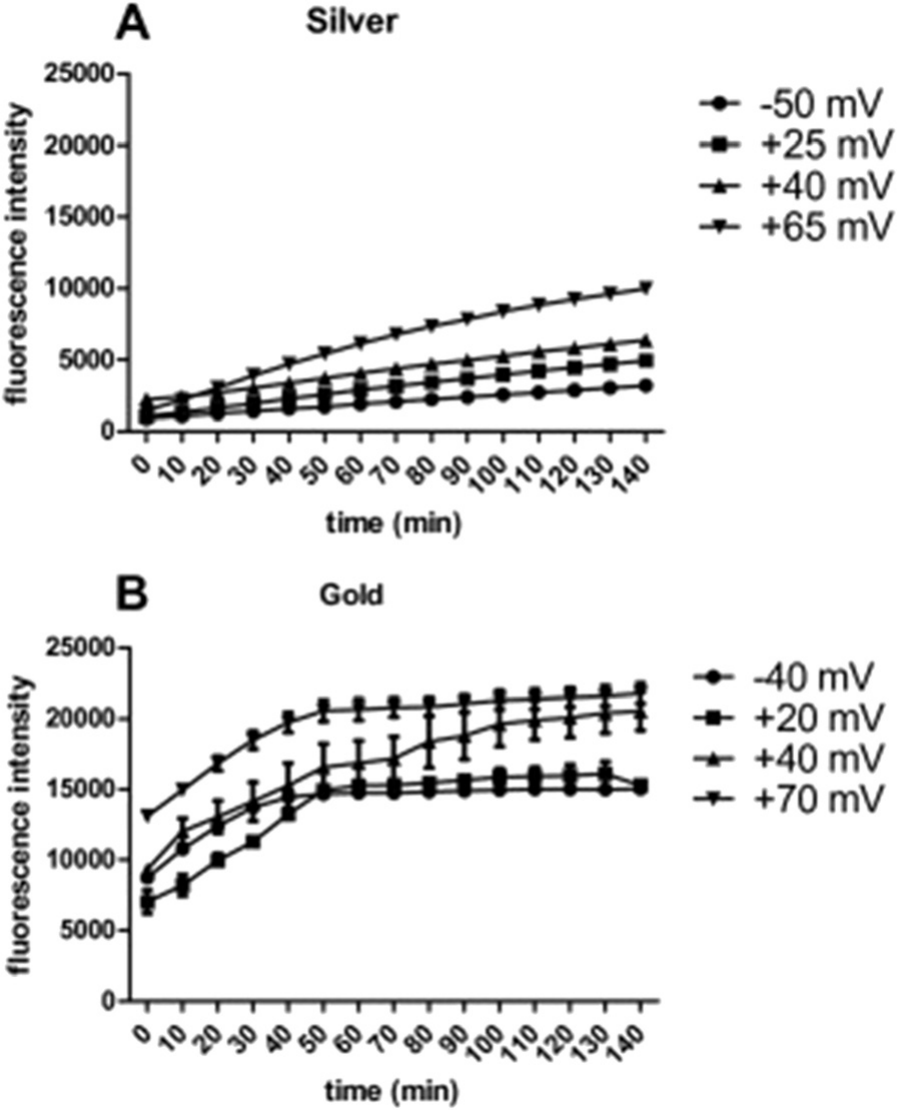
**ROS production of functionalized NPs in a cell-free system. A**. Silver NPs. **B**. Au NPs. Fluorescence was measured at 530 nm (excitation 485 nm) every 10 minutes after an initial incubation period of 15 minutes. Data is displayed as mean ± SEM (n=4).

Since functionalized NPs can produce ROS via interactions on their reactive surface, the production of ROS in the presence of the NPs studied was measured in a cell-free system. As seen in Figure 5, Ag NPs produced only low levels of ROS, but in a functionalization-dependent fashion.

In contrast, all of the Au NPs produced relatively high levels of ROS. The particles with negatively charged functional groups produced the least ROS, whereas an increase in the amount of positively charged surface groups was shown to correlate with an increase in ROS production, with the Au NPs coated with the greatest amount of chitosan (Au-CHIT-H) being the most reactive (Figure 5).

In addition, ROS production of the respective solvents, sodium citrate and three concentrations of chitosan, was determined (Additional file 8). Interestingly, ROS production of the tested solvents appeared to be slightly increased in comparison to that of Ag NPs, yet much smaller compared to that of Au NPs. The trend observed with Au NPs, where an increase in positive charge created by an increase in chitosan concentration resulted in an increase in ROS production, could not be observed. The amount of ROS produced by the solvents was very similar and no increase in ROS production was observed with an increase in chitosan concentration.

#### Effects of biological solutions on ROS production in a cell-free system

**Figure 6:**
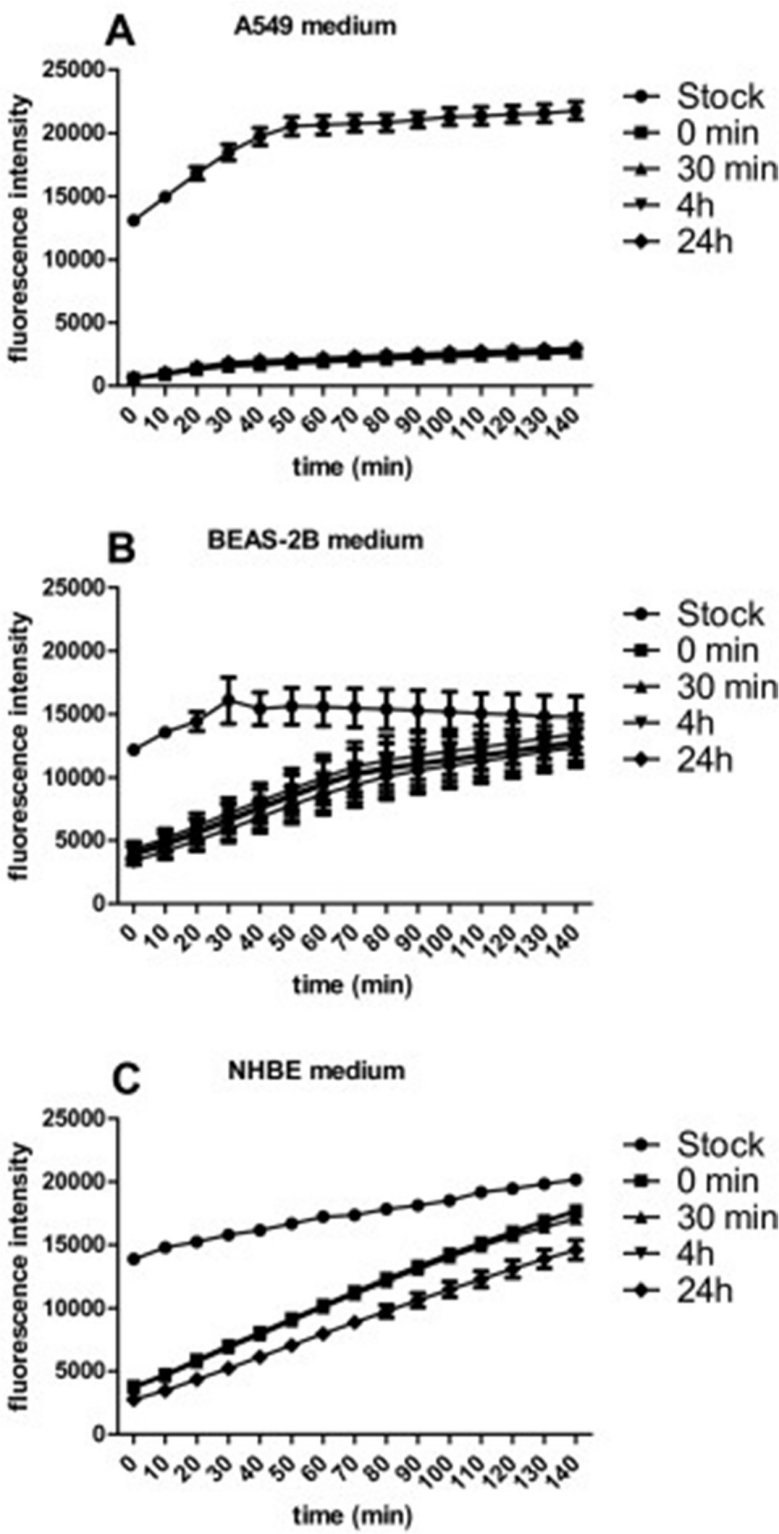
**ROS production of chitosan-coated Au NPs (Au-CHIT-H) in a cell-free system after incubation in cell culture medium. A**. A549 medium. **B**. BEAS-2B medium. **C**. NHBE medium. Fluorescence was measured at 530 nm (excitation 485 nm) every 10 minutes after an initial incubation period of 15 minutes. Means ± SEM of n=3 are shown.

**Figure 7:**
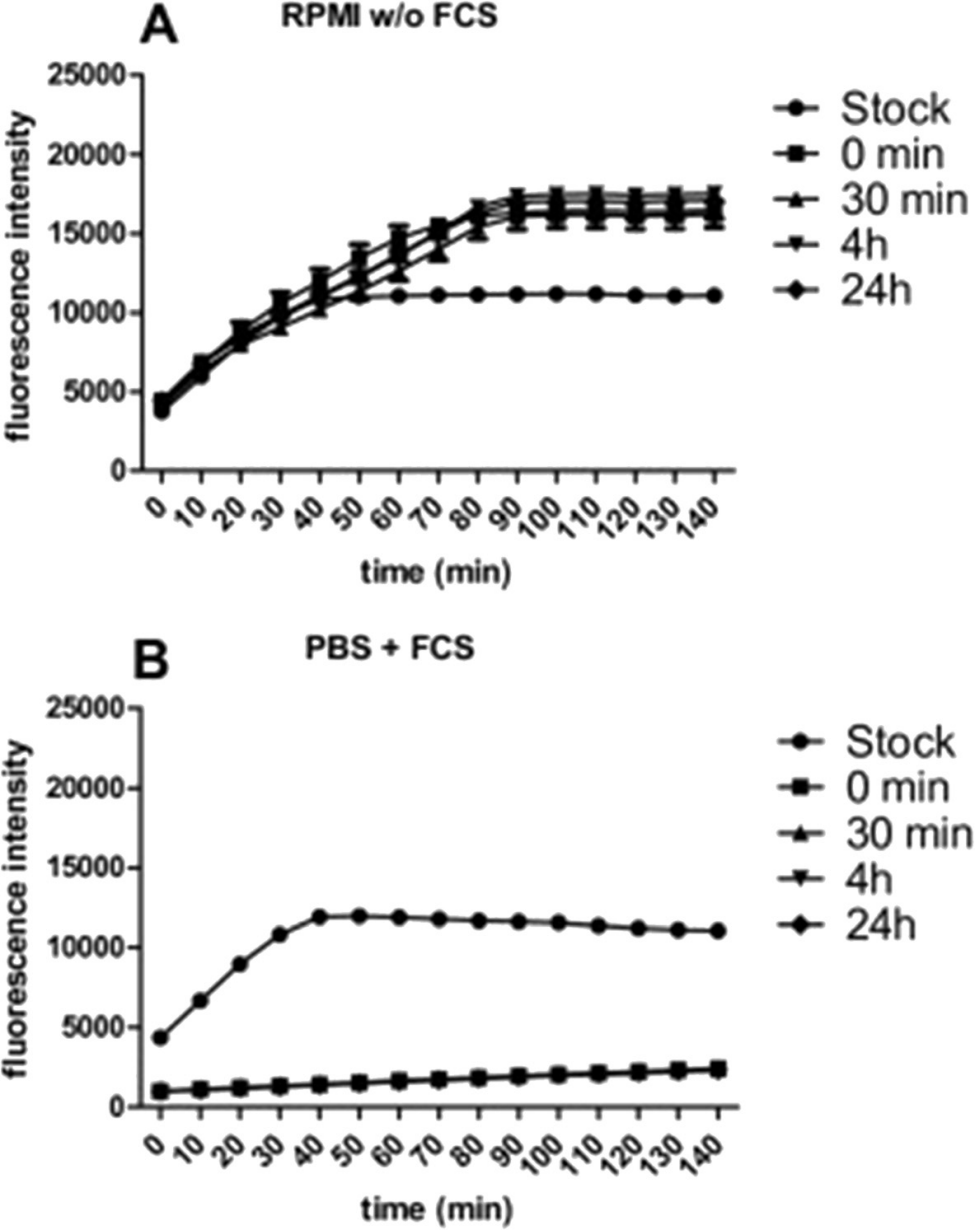
**Effect of FCS on the ROS production of chitosan-coated Au NPs (Au-CHIT-H) in a cell-free system. A**. Incubated in RPMI w/o FCS. **B**. Incubated in PBS + FCS. Fluorescence was measured at 530 nm (excitation 485 nm) every 10 minutes after an initial incubation period of 15 minutes. Means ± SEM of n=3 are shown.

As previously mentioned, the protein corona surrounding NPs may play a crucial role in cellular responses. It is therefore critical to study the NPs in the presence of cell culture media. Differently functionalized Ag and Au NPs were incubated in cell culture media corresponding to the three different cell type-specific media used in this study for different periods of time (0.5, 4 and 24 hours). Au-CHIT-H was chosen as representative class in Figure 6. The most prominent response was seen after NP-incubation with A549 medium, which contains 10% foetal calf serum (FCS) (Figure 6a), where ROS production by particles dropped to a minimal level. This effect was observed across all particles and functionalizations/charges. To further correlate the loss of ROS production with the presence of FCS in the media, NPs were incubated with A549 medium containing no FCS or with PBS containing FCS (Figure 7). PBS containing FCS and not full A549 medium was used to ensure that any observed effects are due to the FCS present in A549 medium and not to any other constituents of the medium. When the particles were incubated with medium containing no FCS (Figure 7a), ROS production did not change. However, when the particles were incubated with PBS containing FCS, ROS production by the particles decreased significantly (Figure 7b). Both, BEAS-2B and NHBE media do not contain serum. However, both media are supplemented with pituitary gland extract. The ROS production of Au-CHIT-H dropped by more than 50% after the incubation in both media (Figure 6b,c), yet was still found to be higher than the ROS production in A549 medium. The data show that ROS production observed in cell cultures derives essentially from the cells used, since the proteins contained in all the growth media effectively abrogate ROS production directly at particle surfaces.

#### Effects of cell culture components on the NPs surface charge

As depicted in Table 3, the surface charge of the NPs used in this study is altered dramatically after incubating the particles in cell culture medium. A 24-hour incubation in A549 cell culture medium resulted in a complete loss of the positive surface charge. This effect was observed in all positively charged Ag and Au NPs. Interestingly, when positively charged NPs where incubated in BEAS-2B medium, the surface charge also dropped significantly, but the positive charge of both Au and Ag NPs were maintained. In contrast, Ag and Au NPs incubated in NHBE medium all carried a negative surface charge after a 24 hour incubation. This effect was also observed when particles where incubated with cell culture media for 4 hours, yet it was less profound (Additional file 9). Here, NPs incubated in BEAS-2B medium carried a much higher positive charge and those incubated in NHBE medium ended up with a very low negative charge as compared to those incubated for 24 hours.

**Table 3:** **Surface charge (N = 3, mV ± SEM) of NPs as synthesised and after a 24 hour incubation in different cell culture media**

**NPs name**	**As synth**	**In A549 medium**	**In BEAS-2B medium**	**In NHBE medium**
Ag-SC	−50 ± 1.4	−27 ± 0.5	−26.2 ± 0.2	−37 ± 1.2
Ag-CHIT-L	+25 ± 0.9	−35 ± 0.9	+10.3 ± 0.4	−26.6 ± 0.6
Ag-CHIT-M	+43 ± 0.4	−25 ± 0.7	+9.8 ± 0.2	−34 ± 0.9
Ag-CHIT-H	+60 ± 0.9	−14.4 ± 0.4	+10.2 ± 0.6	−25.7 ± 2.8
Au-SC	−45 ± 0.2	−32 ± 0.5	−24.4 ± 0.2	−25 ± 0.2
Au-CHIT-L	+23 ± 1.0	−33 ± 0.7	+6.8 ± 0.2	−31.9 ± 0.1
Au-CHIT-M	+40 ± 0.6	−33 ± 0.8	+7.9 ± 0.9	−35 ± 0.4
Au-CHIT-H	+65 ± 1.0	−23.3 ± 0.5	+9.0 ± 0.1	−28.0 ± 1.3

## Discussion

The results of the TEER measurements and the tight junction staining demonstrated that the cell types tested had different growth capacities under the settings used in this study. These growth patterns corresponded well to the cytotoxic responses. A549 cells growing in a monolayer, and with well-developed tight junctions, as would be found *in vivo,* were not responsive to any of the particles tested. In contrast, NP exposure did not induce cytotoxicity in NHBE cells, which did not form a monolayer and no tight junctions. BEAS-2B cells did form a multilayer with tight junctions, with cytotoxicity observed in response to high levels of chitosan on functionalized Au NPs.

Another reason for the divergent cytotoxicity data found might be the difference between primary cells and cell lines. Both A549 and BEAS-2B cells are immortalized and might therefore be differently susceptible towards external stimuli. However, these two cell lines are derived using different methods, A549 were created from cancerous cells [42] and BEAS-2B were immortalized using an adenovirus [43]. One might describe the monolayer growth of A549 cells as more natural compared to that of BEAS-2B cells, where cells continue to grow which results in the formation of multilayers. The rapid growth of a cellular monolayer by A549 cells is one reason, why they are most frequently used in lung cytotoxicity studies. Unlike the two cell lines which originate from a single donor before immortalization primary NHBE cells were extracted from different healthy donors [44] by the supplier, so a donor-to-donor variation between cell charges is always given. It has been shown that NHBE cells grown at an air-liquid interface can form a full monolayer with working tight junctions after 8 days [50]. In the present study a confluent monolayer was not obtained; this, and the lower NHBE cell number upon particle exposures, may be the reason for the observed cytotoxicity in these cells that was not observed in the other cell treatments.

It has been reported that NPs composed of chitosan are internalized by cells, e.g. A549 cells

[51-54], yet it is also known, that the uptake of NPs by cells will not automatically result in a cellular response. In fact, chitosan-coated Au NPs have been shown to be taken up by A549 cells [55], and have been reported to be biocompatible [56,57] and for that reason are increasingly used as carriers in drug or gene delivery systems [58,59]. However, Choi *et al*. reported that chitosan-coated AuNPs internalised by A549 cells provoke cell damage through both apoptopic and necrotic pathways [55]. The NPs used by Choi *et al*. are of similar nature to those used in the study here, since they are also chitosan-coated Au NPs, yet are slightly larger, 17 nm compared to 7 nm in our study. The positive surface charge of their NPs, as determined by zeta-potential measurements, is comparable to that of the Au-CHIT-M presented here (~40 mV). The size difference of the particles used by Choi *et al*. does not allow to presume that our NPs would also be internalised, but the similarities in surface charge does infer that similar cell interactions would have occurred. As cell death was not observed in response to the comparable AuNPs (Au-CHIT-M) it can be assumed that the cytotoxicity of the AuNPs used by Choi *et al*. was due to the increase in NP size, or more likely due to the increased concentration which cells were exposed to.

Other studies have shown that chitosan can induce cytotoxicity in other cell types [51,52], but high levels of chitosan were used in these studies resulting in the observed cytotoxicity. Since the core of both Ag and Au NPs will stabilize the chitosan on the surface during synthesis, thereby forming robust conjugates [53], the amount of free chitosan used in our study will presumably be lower compared to those amounts in the studies mentioned above as no additional chitosan will be released by the NPs [54]. Thus, the observed cellular effects are more likely to be due to the chitosan-coated NPs and not due to the chitosan itself [55].

Possible differences in the cellular uptake of the NPs by the three cell types used in this study might provide an additional explanation to the divergent cellular responses [56], which has been previously compared [57]. Cellular uptake studies will therefore be performed as part of a future study.

Differently charged Ag and Au NPs were chosen for this study on purpose, as it is becoming increasingly evident that the surface charge of NPs is a crucial characteristic of NPs. Au NPs of similar sizes and surface charges (+45 mV, −41.5 mV) ,yet with a different surface coating (AUT, peptidic biomolecules) to those presented here were analysed by Ojea-Jiménez *et al.* [60]. They reported an increase in cellular uptake of positively charged NPs in comparison to negatively charged NPs. They also reported that once taken up by cells, a large fraction of the positively charged NPs migrated towards the vicinity of the nucleus. Thus, positively charged Au NPs might be more feasible for gene therapies than negatively charged NPs [60]. Similarly, Oh *et al.* reported an increase in cellular uptake with positively charged (+25 mV. +29 mV, +42 mV and + 55 mV) Au NPs in macrophages in comparison to negatively charged Au NPs (−15 mV, −30 mV, − 35 mV and −38 mV) [61]. Furthermore, the surface charge of NPs has been shown to affect the cytotoxicity of NPs. Schaeublin *et al.* reported an increase in toxicity in the human keratinocyte cell line HaCaT following the exposure to positively and negatively charged Au NPs, whereas neutral Au NPs were less harmful [62]. Others come to the conclusion that cationic can be considered to be more toxic than anionic NPs in red blood cells and COS-1 kidney cells [63]. Unfortunately, the latter study does not specify the surface charge of their NPs.

Our data demonstrated that an increase in chitosan coating affected the response to Au NPs more than to Ag NPs. The lower stability of the chitosan-Ag NPs conjugate as compared to the Au NPs conjugate could explain these findings. The chitosan molecules are not only attached more stably to the surface of the Au NPs, but also more densely [64,65]. Thus, the positive surface charge of Ag NPs decreased faster under cell culture conditions compared to Au NPs (observed during the four incubation of both NPs), as they will be subject to a greater amount of oxidation at their surface. This oxidation will weaken the interaction between the chitosan layer and the NP surface thereby reducing the compactness and robustness of it during exposure. This reduction in positive surface charge of Ag NPs may result in a less dense interaction of Ag NPs with cells compared to Au NPs, which may provide an explanation as to why Au NPs were found to induce greater membrane impairment than Ag NPs.

It is known that the protein corona of NPs may greatly affect their influence on cells [27]. Each of the cell types used in this study was cultured in a different medium. A549 cells were cultured in RPMI containing 10% FCS. Both BEAS-2B and NHBE cells were cultured in serum-free medium, however, other proteins, such as pituitary gland extract, were present in these media. It has previously been reported that the surface charge of NPs affects the composition of the protein corona formed [58] and that serum proteins present in solution will in turn affect the resulting surface charge of the NP-protein complex [59]. The present study shows that pre-incubation of NPs in the above-mentioned cell culture media affected the surface charge of the NPs, which influenced the cellular responses.

All NPs used in this study, irrespective of their core or surface functionalization, lost their positive surface charge following a 24 hour incubation in A549 medium. Our data suggests that the NPs quickly formed a protein corona from the FCS contained in the A549 medium, as even short incubation periods resulted in a negative charge. The new surface charge of the NPs can be considered to be the average of the surface charge of the FCS proteins that adsorbed onto the surface of the NPs. The adsorption process of the proteins onto the NPs is highly dependent on the affinity of the proteins towards the NPs surface [66]. The newly formed protein layer upon the NPs thus covers the original surface coating, thereby masking the original surface charge. We have previously shown that a nearly complete hard corona will surround the NPs after 24 hours [67], thus any observed effect suggests an impact of the protein corona. In contrast, all NPs incubated in medium without FCS, such as the BEAS-2B medium, retained their positive charge after 24 hours. They still adsorbed negatively charged proteins from the medium, as can be seen by the drop in surface charge, indicating the formation of a protein corona. However, the change in surface charge was not as big as the one observed when incubating in A549 medium, since lower amounts of protein are present. NPs incubated in NHBE medium behaved very similar to those incubated in BEAS-2B medium. BEAS-2B and NHBE medium are very similar in their composition, Amphotericin-B is found only in the NHBE medium. Although the exact concentrations of the supplements were not disclosed by the supplier, one can postulate that the difference in surface charge after incubation was due to concentration differences between the media. Notably, the NPs coated with the highest amount of chitosan ended up with the lowest negative charge of all NPs used.

All of the cells reacted to exposures of Au NPs coated with a large amount of chitosan, thereby carrying a high positive surface charge after synthesis. Since these were the NPs that maintained the least negative charge in A549 and NHBE medium and even remained positive in BEAS-2B medium, it is suggested that their initial high positive charge is directly affecting the observed cytotoxicity. Different surface charges may affect various parameters, including amount and type of proteins and other biological compounds, corona hardening, and intensity as well as route of uptake into cells. Dissecting these interesting mechanistic aspects was beyond the scope of the present study.

Several studies have investigated NP-induced oxidative stress in cells [68-70]. Normally, ROS is only generated to a low extent in healthy cells and is quickly detoxified by the cells antioxidant defence mechanisms (mainly glutathione and antioxidant enzymes). However, an imbalance between ROS and defence mechanisms results in oxidative stress [22]. In the present study, functionalized NPs carrying a positive surface charge appear to induce a higher amount of ROS within the cells. The highest production of ROS following exposure to NPs was found in A549 cells. ROS production was increased in both BEAS-2B and NHBE cells, yet not to the same extent. These findings are in line with a study by Ekstrand-Hammarström *et* al. who analysed the effects of titanium dioxide NPs on A549, NHBE and BEAS-2B cells [57]. Similar to the results presented here, the group finds the least amount of NPs induced ROS in NHBE cells compared to the other two cell types, which might be due to different uptakes rates of NPs in the three cell types. Since uptake studies were beyond the scope of the study presented here, one can only postulate that the differences in ROS production are a result of the differences in cellular uptake, which will be the subject of a future study.

In order to understand the production of ROS with the characteristics of the NPs used in this study and to obtain information on the oxidative capacity of the NPs, ROS measurements in a cell-free system were performed. In this system, Ag NPs, irrespective of the surface charge, only produced very small amounts of ROS. In contrast, all of the Au NPs produced large amount of ROS, where the amount of ROS produced increased in correspondence with the surface charge. It is interesting that the oxidative capacity of, presumably neutral, Au was in this study found to be more detrimental than Ag, even though the latter is widely used as bacteriostatic agent due to its toxic potential [60].

Even though results on both cell-mediated and cell-free ROS production of the NPs used in this study were compared, no direct link between these two parameters could be detected. However, the results allow a better understanding of particle-mediated ROS production, in cell-free and cellular systems. It has previously been shown that the oxidative capacity of NPs can influence their ability to induce oxidative stress, but recently published data show that this might not always be the case. For example, a study performed by Weissenberg *et al.* showed that intracellular ROS production still occurred after extracellular ROS produced by NPs was blocked using an inhibitor [71]. Furthermore, in the study presented here, extracellular ROS production was inhibited through binding of cell culture medium proteins to the NPs, while intracellular ROS production in response to the same particles was observed within cellular exposures.

Further experiments have shown that proteins adsorbed to the NPs surface reduced the amount of ROS being produced. During cell culture experiments, the NPs will encounter a vast amount of proteins. The amount and type of proteins is dependent on the cell culture medium used. In this study, we demonstrated that the widely used RPMI medium supplemented with 10% FCS greatly affects the ability of NPs to produce ROS. Even short incubation times in the medium resulted in a complete loss of ROS production by the particles in a cell-free system. Some ROS production was still observed in A549 cells, even though the cells presumably only interacted with cells coated with FCS. The loss of ROS production might be explained with the formation of a protein corona consisting of serum albumin. Albumin has been reported to have anti-oxidant properties [61]. Izak-Nau *et al.* used MALDI-TOF to confirm that the main component of the protein corona surrounding charged NPs is bovine serum albumin [59]. It is therefore likely, that ROS production by NPs is blocked once a protein corona consisting of albumin is formed. Further studies on protein-NPs interaction are needed to provide additional evidence for this.

Additional research will be needed to fully understand how the NPs affect the cells with respect to the protein corona of differently functionalized NPs in different biological media, how this affects cellular uptake of NPs and how intracellular ROS production is linked to the oxidative capacity of NPs.

## Conclusion

Several conclusions can be drawn from the results of this study.

First, the type of lung epithelial cells used to analyse the effects of NPs greatly affects the results. The properties of the cell type used need to be considered for correct interpretation of data. By understanding the growth characteristics of different cell types and how a particle effect can be different between these cell types, an improved design of *in vitro* systems can be supported.

A surface charge of +30 to +40 mV might be considered as harmless under cell culture conditions, while a surface charge above +60 mV has to be designated as problematic and it can be postulated that inhalation of highly charged wet nanoaerosols, as may be stably produced in nature (water falls) or during nanomedical approaches by nebulization/atomization from NP suspensions [62,63], may carry serious risks.

Finally, this study supplies evidence towards the impact of the components of cell culture medium, mainly FCS, on the characteristics of NPs. FCS will not only reduce the surface charge of NPs, but will also affect their ability to produce ROS.

While the data allow conclusions about safety studies *in vitro* with widely used cell culture models, the behaviour of lung tissue under physiological conditions needs to be verified with other approaches. It can be predicted that the biological compounds present will play a major role in defining which types of NPs elicit cell damage through oxidative stress and that, based on cell culture models, high chitosan coating conferring strong positive surface charge may be a risk factor.

## Materials and methods

### Cells

The adenocarcinomic human alveolar basal epithelial cell line (A549) was maintained in RPMI 1640 medium (PAA Laboratories GMBH, Pasching, Austria) containing 10% FCS, 5% penicillin/streptomycin and 5% L-glutamine. The human bronchial epithelial cell line BEAS-2B, originally isolated from a non-cancerous patient and immortalised by an adenovirus 12-SV40 hybrid, was grown in LHC-9 basal medium containing supplements. Normal human bronchial epithelial cells NHBE (CC-2540, Lonza, Basel, Switzerland), isolated from healthy donors, were cultured in bronchial epithelial growth medium (BEGM) supplemented with BEGM® Single quots® (Lonza, Basel, Switzerland). All cells were sub-cultured by removing the cell culture medium from the cell culture flasks and washing the cells with 2 ml PBS. After removing the PBS, 2 ml of pre-warmed trypsin was added and the cells were incubated at 37°C for 2–3 minutes until all cells detached from the flask, observed via light microscopy. To stop the trypsin reaction, 8 ml pre-warmed cell culture medium was added to the flask. The number of living cells was determined via trypan blue exclusion and counted within a haemocytometer. The cells were plated on either 24-well or 96-well plates (Corning Inc., city) at a density of 1 × 10^5^ cells/ml, where 1 ml of medium was added to a well of a 24-well plate and 100 μl to a well of a 96-well plate. A549 cells were grown for 4 days, BEAS-2B and NHBE cells were grown for 7 days, ensuring that the cells reached a confluent monolayer, before they were exposed to the NPs.

Cells were stored in liquid nitrogen and, after thawing, maintained at 37°C and 5% CO_2_. Cells were never cultured for more than one month and the NHBE cells were only frozen and thawed once. The presence of mycoplasma was determined once a week using the MycoAlert™ kit (Lonza, Basel, Switzerland) and infected cultures were disposed of immediately.

### Transepithelial electrical resistance measurements

In order to assess the integrity of the epithelial layer of A549, BEAS-2B and NHBE cells, transepithelial electrical resistance (TEER) was measured using a TEER electrode (WPI, Sarasotay, USA). Cells were grown on a 24-well plate containing well inserts (Millipore Corporation, Billerica, MA) with a pore size of 0.4 μM in diameter. TEER values were measured every 24 hours. Before each measurement, the medium was changed and the electrode was washed with RPMI medium between measurements. The TEER value was calculated for the dimension [Ohm*cm^2^] by subtracting the medium only control from the measured value and then multiplying it by the surface area of the insert (0.33 cm).

### Tight junction staining

A549, BEAS-2B or NHBE cells were grown on 24-well cell culture plates and tight junction staining was performed for each day after an initial growing phase of 24 hours. Cells were fixed with 3.7% formaldehyde in PBS for 10 minutes at RT and washed twice with 500 μl PBS. Following the fixation, 250 μl 1× saponin in PBS was added at RT for 5 minutes and again washed twice with 500 μl 1× PBS. The primary antibody (5 μl rabbit-anti-human claudin-1 in 250 μl saponin/PBS) was added and incubated for 45 minutes in the dark. The cells were washed with 1 ml saponin/PBS before the secondary antibody (2 μl goat-anti-rabbit IgG-PE in 250 μl saponin/PBS) was added. The plate was then incubated for 30 minutes in the dark, cells were washed with 500 μl 1× PBS. Fluorescence micrographs were taken using a fluorescence microscope (Olympus I × 70-S1F, Austria) using a 10× objective.

### Nanoparticles

#### Synthesis

Nanoparticle preparation was performed following the most common synthesis recipes in water with some modifications to achieve the desired characteristics regarding size and surface charge. All reagents were purchased from Sigma-Aldrich (St. Quentin Fallavier, France) and used as received. All glass material was sterilized and depyrogenated in an oven prior to use.

#### Gold Nanoparticles (Au NPs)

Citrate-coated 10 nm Au NPs were obtained with a procedure based on Turkevich et al. [72] consisting of the fast injection of 1 mL of a solution of hydrogen tetrachloroaureate (HAuCl_4_) 25 mM to a boiling solution containing trisodium citrate (SC) at 2.2 mM under vigorous stirring. After 3 minutes, when the suspension acquired the characteristic red colour of the colloidal gold, it was cooled down to room temperature (RT). NPs were loosely coated with the negatively charged citrate ions.

*Chitosan-coated Au NPs of 7–10 nm mean diameter* were obtained were obtained by a variation of a procedure based on the synthesis described in Jana *et al*. [73]. In brief, an aqueous solution containing as precursor HAuCl4 at 2.5 × 10^−4^ M and chitosan (0.001%, 0.01%, 0.1% (w/v) was prepared. To this solution, 1 ml of ice-cold 0.1 M NaBH4 was added under constant stirring. Increasing concentrations of chitosan were used as capping agent conferring a range of different positive surfaces charge to the Au NPs.

#### Silver Nanoparticles (Ag NPs)

*Citrate-coated 10 nm Ag NPs.* 5 mL of trisodium citrate 0.1 M were injected to a boiling solution of 50 mL of silver nitrate (AgNO_3_) 1 mM and left under vigorous stirring for 5 minutes. The resulting solution was cooled down in another vial to avoid deposition of silver on the glass surface. Citrate ions were the coating agent as in the case of Au NPs. An overview of the NPs synthesized for this study can be viewed in Table 4.

**Table 4:** **Overview of the NPs used in this study**

**NPs name**	**Size (nm)**	**Surface coating**	**Ζ-potential (mV)**	**NPs/ml**	**mg/ml**
Ag-SC	10 ± 2.0	Sodium citrate	−50 ± 1.4	1 × 10^12^	0.027
Ag-CHIT-L	10 ± 4.0	Chitosan 0.001%	+25 ± 0.9	5 × 10^12^	0.027
Ag-CHIT-M	10 ± 4.0	Chitosan 0.01%	+43 ± 0.4	5 × 10^12^	0.027
Ag-CHIT-H	10 ± 4.0	Chitosan 0.1%	+60 ± 0.9	5 × 10^12^	0.027
Au-SC	10 ± 1.5	Sodium citrate	−45 ± 0.2	3 × 10^12^	0.032
Au-CHIT-L	7 ± 3.0	Chitosan 0.001%	+23 ± 1.0	5 × 10^12^	0.025
Au-CHIT-M	7 ± 3.0	Chitosan 0.01%	+40 ± 0.6	5 × 10^12^	0.025
Au-CHIT-H	7 ± 3.0	Chitosan 0.1%	+65 ± 1.0	5 × 10^12^	0.025

The designation of NP name used in this table is used throughout the text. NPs size was determined by determining the size distribution of NPs in TEM images (N = 3, ±SEM). Zeta potential was measured in the colloidal solutions of the NPs after synthesis at a pH of 7 for the citrate coated NPs and a pH of 5 for the chitosan coated NPs.

### Nanoparticle characterization

**Figure 8:**
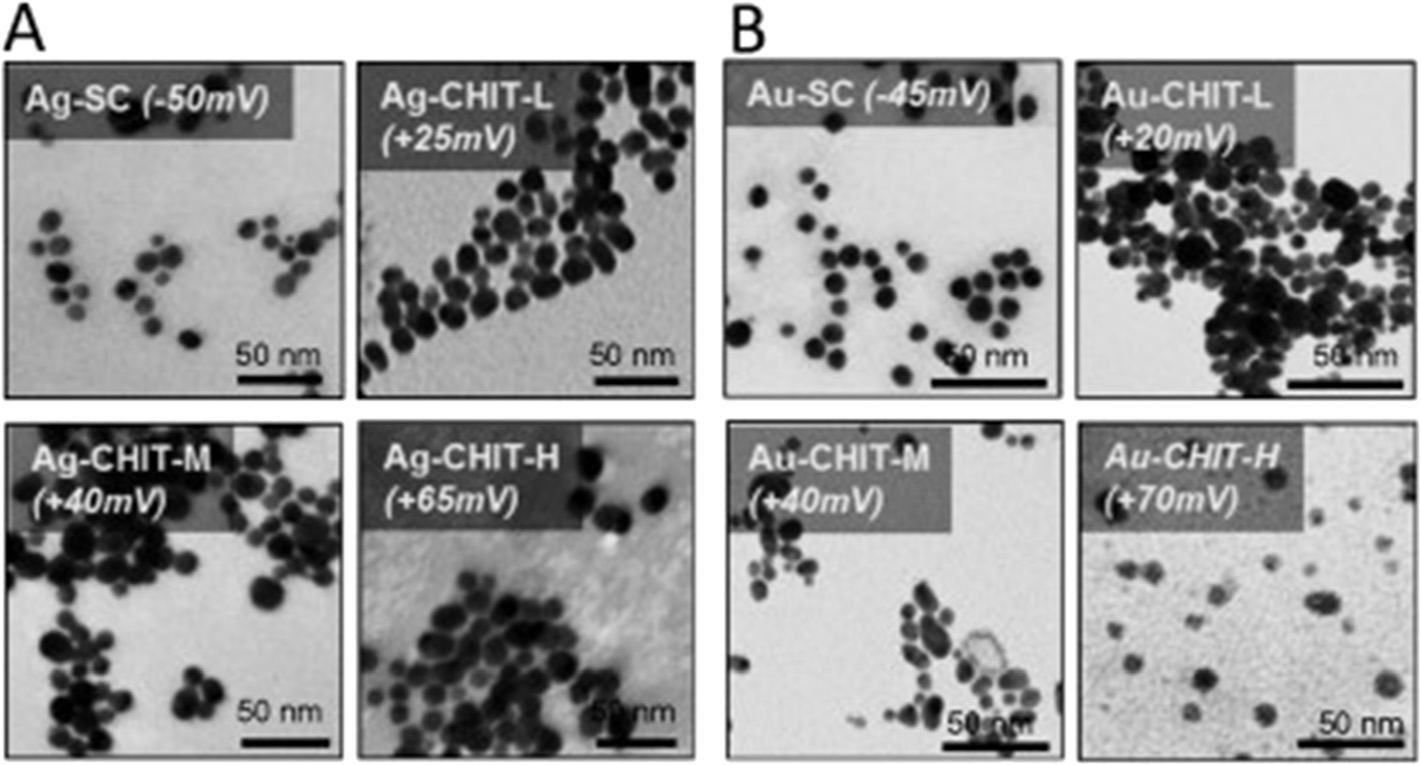
**TEM images of sodium citrate or chitosan coated Ag (A) and Au (B) NPs after synthesis.**

NPs were characterised as previously described by Casals *et al.* [27]. Briefly, the NPs sizes were determined by TEM measurements using a JEOL 1010 electron microscope (Jeol, Tokyo, Japan) at an accelerating voltage of 80 kV on carbon coated cooper TEM grids. A minimum of 1000 particles were computer-analysed and measured to obtain a size distribution (Figure 8). NPs surface charge was determined by zeta potential measurements using a Malvern ZetaSizer Nano ZS (Malvern Instruments, Malvern, UK) operating at a light source wavelength of 532 nm and a fixed scattering angle of 173°. Measurements were performed in the colloidal NPs solution after synthesis, with a pH of 7 for the negatively charged sodium citrate coated NPs and with a pH of 5 for the positively charged chitosan coated NPs.

### Exposure conditions

NPs dispersed in the corresponding synthesis solvents were used in this study at different concentrations. Prior to the exposure of cells to the NPs, a serial dilution was performed to obtain the desired concentrations which ranged from 0.05 to 0.8 μg/cm^2^. These concentrations were calculated as administered dose and not as delivered dose. This is the amount of NPs added to a cell culture well in correlation to the total surface area of the well, referred to as the administered dose. These administered concentrations expressed as μg/cm^2^ convert to 6.25 × 10^12^, 1.25 × 10^12^, 2.5 × 10^12^ and 5 × 10^12^ NPs/ml. For the analysis of cytotoxicity (CTB, LDH), cells grown in 96-well plates were exposed to the NPs for 4, 24, 48 or 72 hours. ROS production was measured in 24-well plates. Here, the cells were exposed to NPs for up to 4 hours.

### Cytotoxicity assays

Two different assays were performed to determine the viability of the cells. The viability of the cell is affected by various factors, one of which is cytotoxicity. One method to determine cytotoxicity is to measure the integrity of the cell membrane. When the membrane is disrupted and damaged, which can happen as a result of exposure to NPs, necrosis occurs and LDH is released into the supernatant. Measuring the amount of LDH is therefore a good parameter for determination of cell membrane integrity. To complement this, the CTB assay which measures the viability of the cells by their metabolism and also refers to the proliferation of the cells, was used as a second cytotoxicity test. Taken together, the results of these assays supply a clear picture of the cells well-being.

#### In house lactate dehydrogenase assay

For the determination of membrane integrity, a modified lactate dehydrogenase assay was used [64]. In brief, 50 μl of a solution containing 1 mg/ml NADH and 0.75 mM pyruvate was added to 10 μl test supernatant. The supernatants were incubated for 37°C for 30 minutes after which 50 μl of 2,4-dinitrophenylhydrazine dissolved in 1 M HCl was added to all wells and the plate was incubated at room temperature (RT) for 20 minutes. Finally, 50 μl of 4 M sodium hydroxide (NaOH) was added and the absorbance at 540 nm was measured using a Tecan plate reader (Tecan infinity 200 pro, Tecan, Maennedorf, Switzerland) after leaving the plate to incubate for 5 minutes at RT.

#### CellTiter-Blue ® (CTB) assay

Cells were exposed to the particles as mentioned above. After exposure, cell viability was determined using the CTB cell viability kit (Promega, Madison, USA). For all tests performed, untreated cells were used as a negative and 0.1% Triton X-100 treated cells were used as a positive control. Fluorescence was measured at 590 nm upon excitation at 560 nm using a plate reader (Infinity 200 Pro, Tecan, Groedig, Austria).

#### Interference control for LDH and CTB assays

To control for interference of NPs within the detection of LDH released during exposure of A549 cells, cells were seeded in the same fashion as previously described. Au and Ag NPs were added at all the concentrations used throughout this study and left for 4 hours, after which Triton X-100 (0.1%) was added to all wells for 10 minutes to induce cell lysis. Control cells were treated with medium only. After this stimulation, the supernatant was removed and centrifuged at 25000 × g to remove NPs, and the LDH assay was performed as previously described. This would enable detection of LDH bound to the NPs and therefore removed during centrifugation, which would result in false negative results. These control experiments were conducted with one biological replicate.

To determine if NPs interfere with either the optical readout of the CTB assay, or with any of the assay components, A549 cells were seeded at different cell densities (2 × 10^5^, 4 × 10^5^, 6 × 10^5^, 8 × 10^5^, 1 × 10^6^), left to adhere for 4 hours and then exposed to Au and Ag NPs at all concentrations used in this study and to medium only. The CTB reagent was then added and the protocol followed as previously described. The use of different cell densities would allow the determination of whether present NPs interfere with the CTB reagent, or with the fluorescence readout, as there would be a deviation from each respective medium only control cell population. These control experiments were conducted with one biological replicate.

### Detection of reactive oxygen species

#### DCFH-DA assay for cellular ROS production

Cell-mediated reactive oxygen species production was detected by a carboxy-dichlorofluorescein diacetate (carboxy-DCFH-DA) assay. This assay was carried out according to the manufacturer’s instructions (Sigma-Aldrich, St. Louis, USA) [65]. Briefly, 5 μl carboxy-DCFH-DA (1 mM) was added to each well of a 24-well plate for 1 hour exposures. For longer exposures, 5 μl carboxy-DCFH-DA was added 60 minutes before the end of the exposure period. Cells exposed to cell culture medium only acted as negative controls, 500 μM H_2_O_2_ was used as a positive control. At the end of the exposure period, cells were washed twice with 500 μl phosphate buffer saline (PBS) and harvested using 75 μl Trypsin. 425 μl cell culture medium containing FCS was used to neutralize the trypsin reaction. Cells were then immediately analysed using a flow cytometer (FACSCanto™, Becton Dickinson). All steps, including the flow cytometry assessment, were carried out with minimal light to avoid effects caused by the light sensitivity of the dye.

#### *Cell-free* ROS production

DCFH-DA powder was dissolved in ethyl alcohol to prepare a 1 mM stock. 10 ml 0.01 M sodium hydroxide (NaOH) was added to 2.5 ml stock solution and left in darkness for 30 min to deacetylate. Then 487.5 ml sodium phosphate buffer with a pH of 7.2 was added to the solution (final DCFH concentration of 5 μM). Horseradish peroxidase (HRP) was used as the catalyst for the oxidation reaction of DCFH-DA at a concentration of 0.5 units/ml. 200 μl of this dye and 100 μl NPs in solvent were added to the corresponding wells in a 96-well plate and left to incubate for 15 minutes at 37°C in the plate reader before fluorescence was measured using 485 nm excitation and 530 nm emission every 10 minutes for 4 hours. All steps were carried out in minimal light to avoid the analysis of artefacts caused by the light sensitivity of the dye.

### Statistical analysis

Data are displayed as mean (±SEM) and were analysed using GraphPad Prism (GraphPad Software Inc., USA). A minimum of three replicates were performed for each method used. Statistical analysis was performed using a one-way analysis of variance (one-way Anova) and Tukey’s test was used as a post-hoc analysis. *P-values* < 0.05 were regarded as statistically significant. Spearman’s rank correlation was performed to analyse trends observed in the ROS production of cells.

## Additional files

Additional file 1:
**Cell membrane integrity, as measured by an increase in LDH-release, following a 4 h exposure of the different cell lines to Ag and Au NPs. An increase in LDH-release is indicated by a decrease in the membrane integrity.** A549 cells (**A**, **B**; means ± SEM of n = 6), BEAS-2B (**C**, **D**; means ± SEM of n = 3) and NHBE cells (**E**, **F**; means ± SEM of n = 3). P-value * < 0.05. Cells treated with medium only were used as negative control (=100%). Additional file 2:
**Cell membrane integrity, as measured by an increase in LDH-release, following a 48 h exposure of the different cell lines to Ag and Au NPs.** An increase in LDH-release is indicated by a decrease in the membrane integrity. A549 cells (**A**, **B**; means ± SEM of n = 6), BEAS-2B (**C**, **D**; means ± SEM of n = 3) and NHBE cells (**E**, **F**; means ± SEM of n = 3). P-value * < 0.05. Cells treated with medium only were used as negative control (=100%). Additional file 3:
**Assessment of NP interference with the LDH assay.** LDH release was induced using Triton-X-100 in A549 cells, prior to the measurement. Additional file 4:
**Cell viability following a 4 h exposure to charged Ag and Au NPs of A549 cells (A, B; means ± SEM of n = 6), BEAS-2B cells (C, D; means ± SEM of n = 3) and NHBE cells (E, F; means ± SEM of n = 3).** P-values * < 0.05, Cells treated with medium only were used as control (100%). Additional file 5:
**Cell viability following a 48 h exposure to charged Ag and Au NPs of A549 cells (A, B; means ± SEM of n = 6), BEAS-2B cells (C, D; means ± SEM of n = 3) and NHBE cells (E, F; means ± SEM of n = 3).** P-values * < 0.05, Cells treated with medium only were used as control (100%). Additional file 6:
**Assessment of interference of Ag NPs (A, B) and Au (C, D) with the CTB assay.**
Additional file 7:
**ROS production measured using the DCFH-DA assay following a 1 h exposure of A549 cells (A, B, means ± SEM of n = 3), BEAS-2B cells (C, D, means ± SEM of n = 3) and NHBE cells (E, F means ± SEM of n = 4) to functionalized Ag and Au NPs.** Spearman’s rank coefficients were calculated for each NP to assess possible charge dependent increases in ROS production. In addition, the coefficients of the highest concentrations of each NP surface charge were calculated to determine if ROS production was charge dependent. P-values * < 0.05, ** < 0.01. Additional file 8:
**ROS production of the NPs solvents in a cell-free system.** Fluorescence was measured at 530 nm (excitation 485 nm) every 10 minutes after an initial incubation period of 15 minutes. Means ± SEM of n = 3 are shown. Additional file 9:
**Surface charge (mV) of NPs as synthesised and after a 4 hour incubation in different cell culture media.**
