# Supplementary figures and images for: The oxidative potential of differently charged silver and gold nanoparticles on three human lung epithelial cell types
Source: J Nanobiotechnology. 2015 Jan 16;13:1. doi: 10.1186/s12951-014-0062-4 (PMC4304186; doi:10.1186/s12951-014-0062-4)

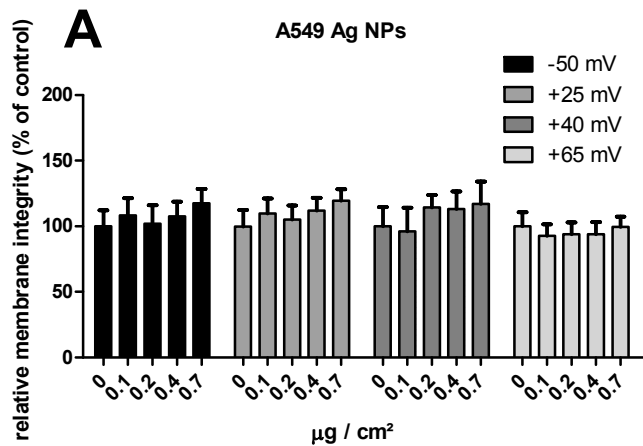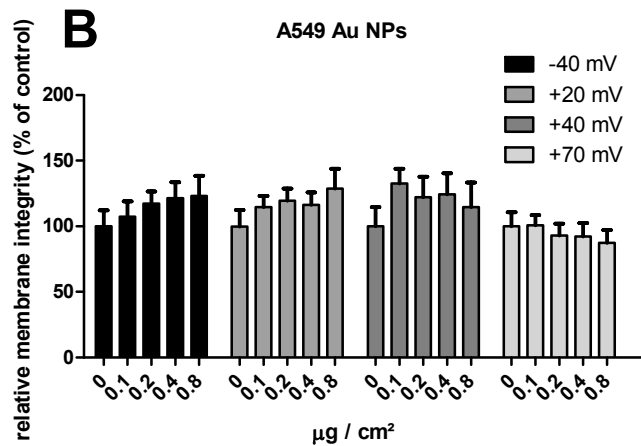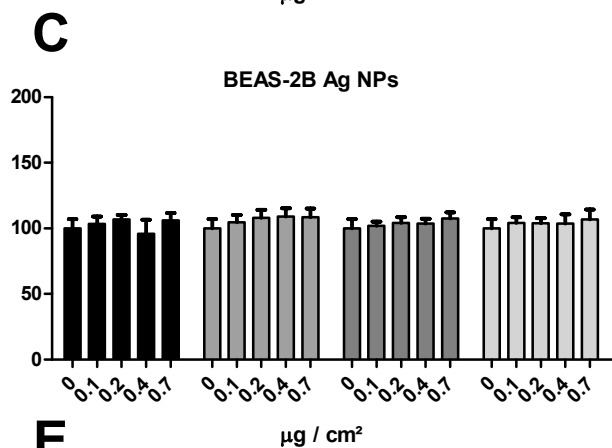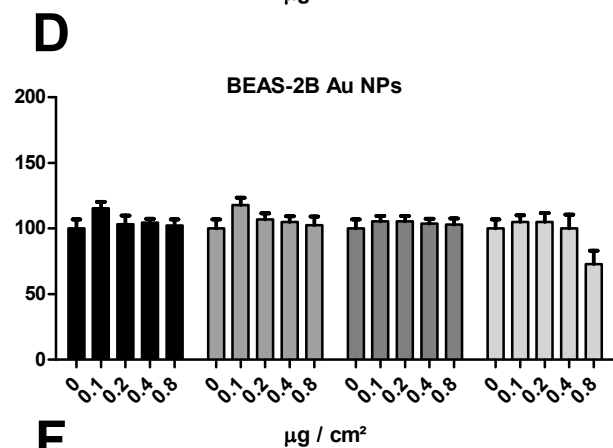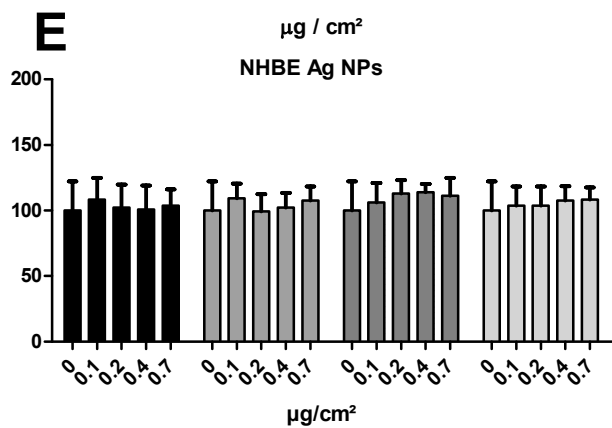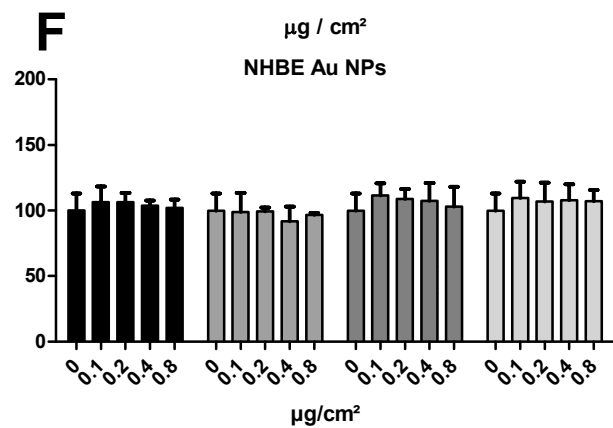

Supplement: Additional file 1: — Cell membrane integrity, as measured by an increase in LDH-release, following a 4 h exposure of the different cell lines to Ag and Au NPs. An increase in LDH-release is indicated by a decrease in the membrane integrity. A549 cells (A, B; means ± SEM of n = 6), BEAS-2B (C, D; means ± SEM of n = 3) and NHBE cells (E, F; means ± SEM of n = 3). P-value * < 0.05. Cells treated with medium only were used as negative control (=100%). [file 12951_2014_62_MOESM1_ESM.pdf]

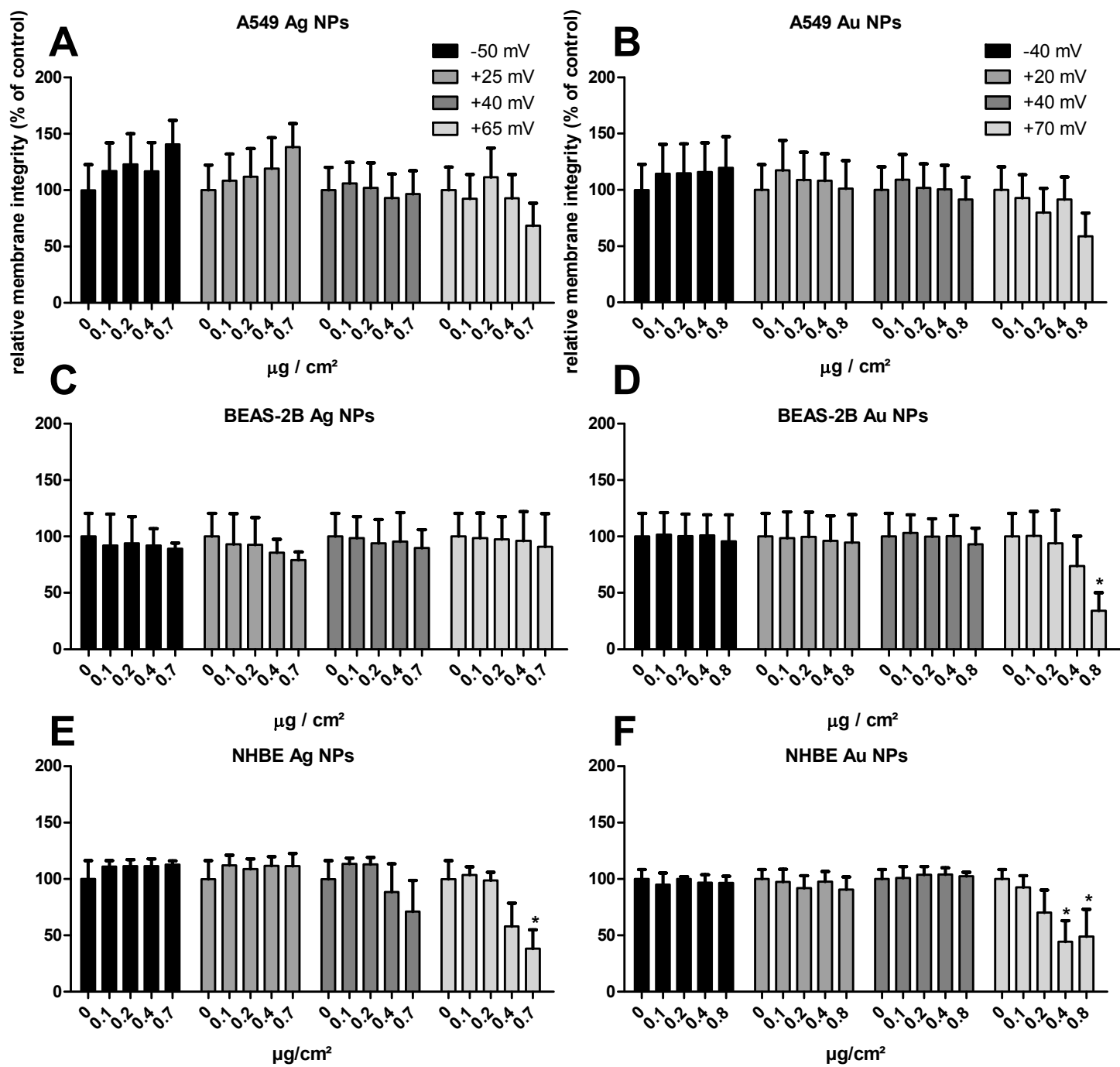

Supplement: Additional file 2: — Cell membrane integrity, as measured by an increase in LDH-release, following a 48 h exposure of the different cell lines to Ag and Au NPs. An increase in LDH-release is indicated by a decrease in the membrane integrity. A549 cells (A, B; means ± SEM of n = 6), BEAS-2B (C, D; means ± SEM of n = 3) and NHBE cells (E, F; means ± SEM of n = 3). P-value * < 0.05. Cells treated with medium only were used as negative control (=100%). [file 12951_2014_62_MOESM2_ESM.pdf]

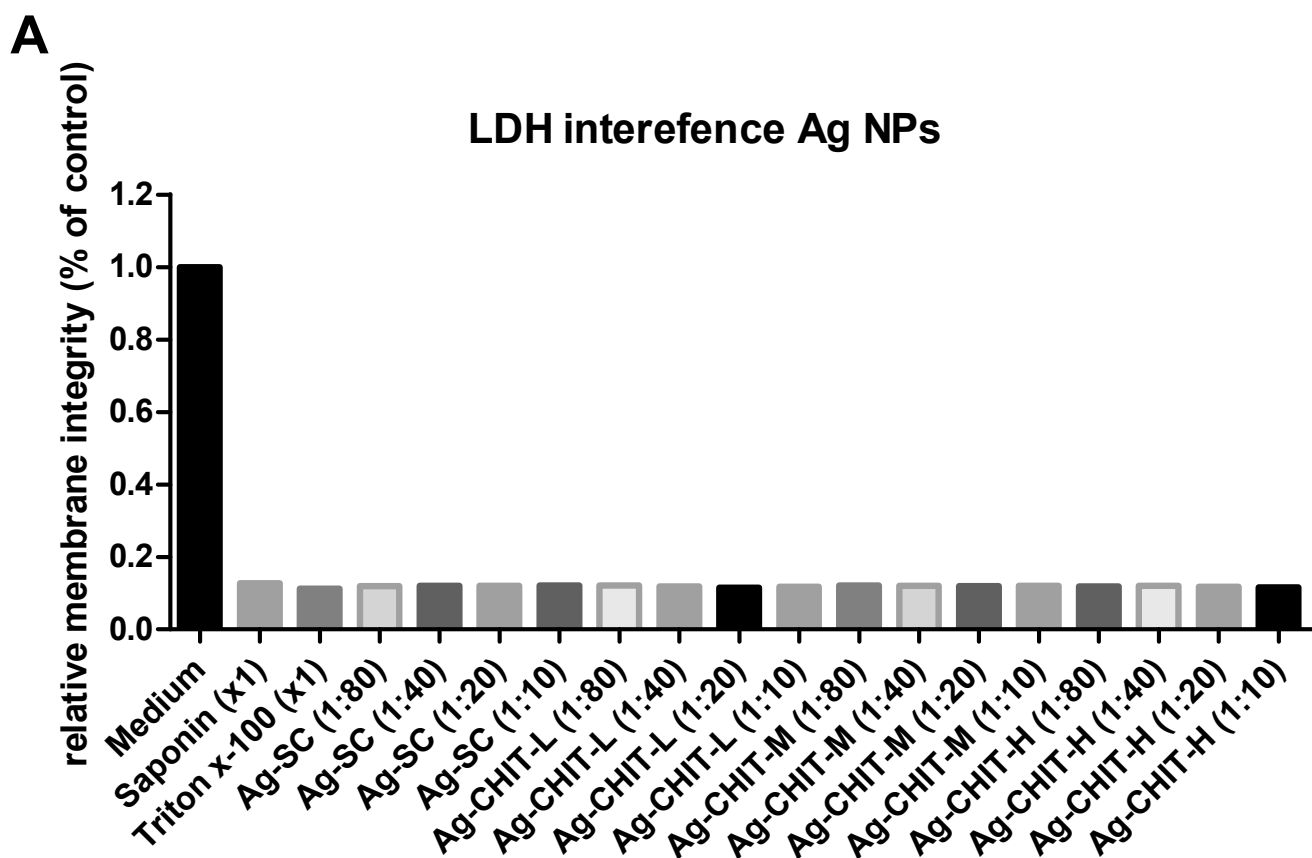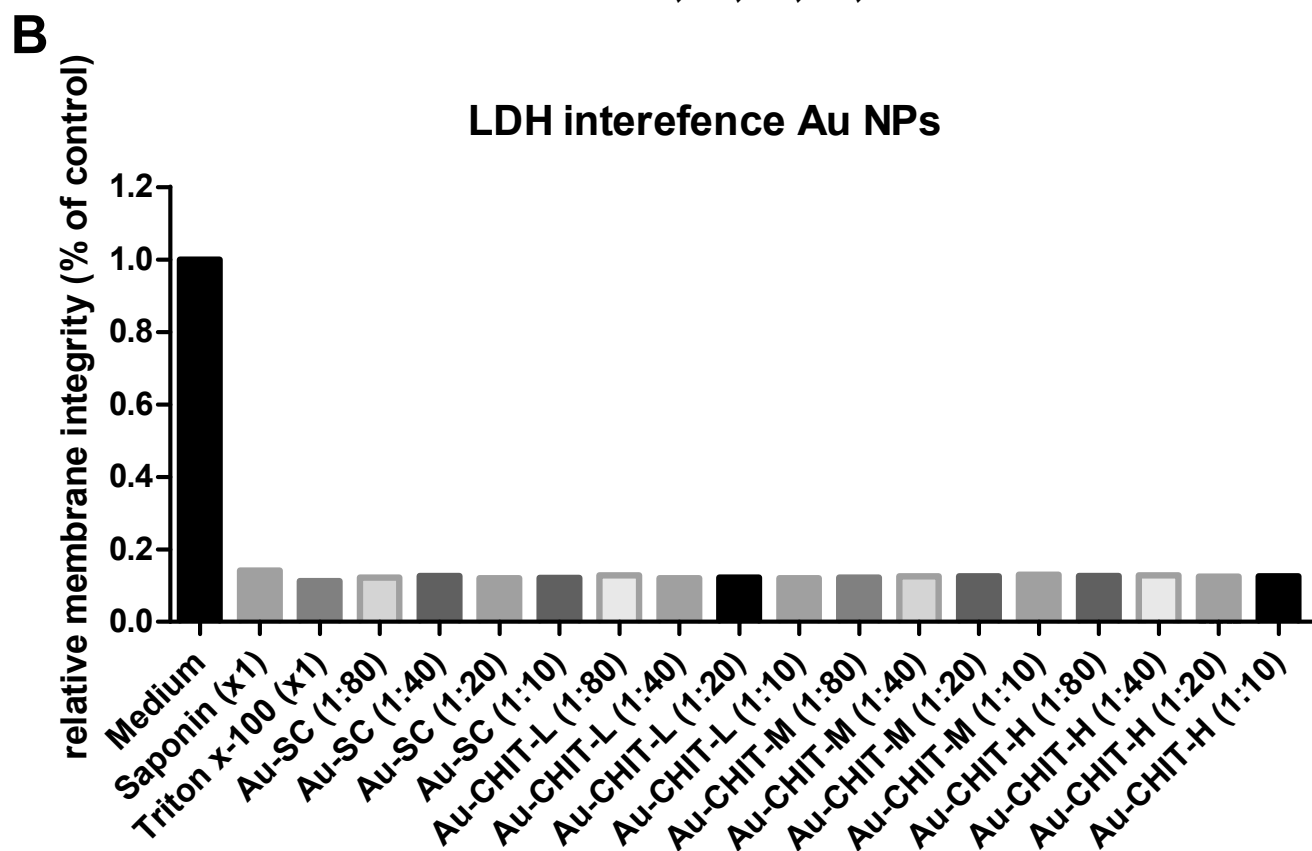

Supplement: Additional file 3: — Assessment of NP interference with the LDH assay. LDH release was induced using Triton-X-100 in A549 cells, prior to the measurement. [file 12951_2014_62_MOESM3_ESM.pdf]

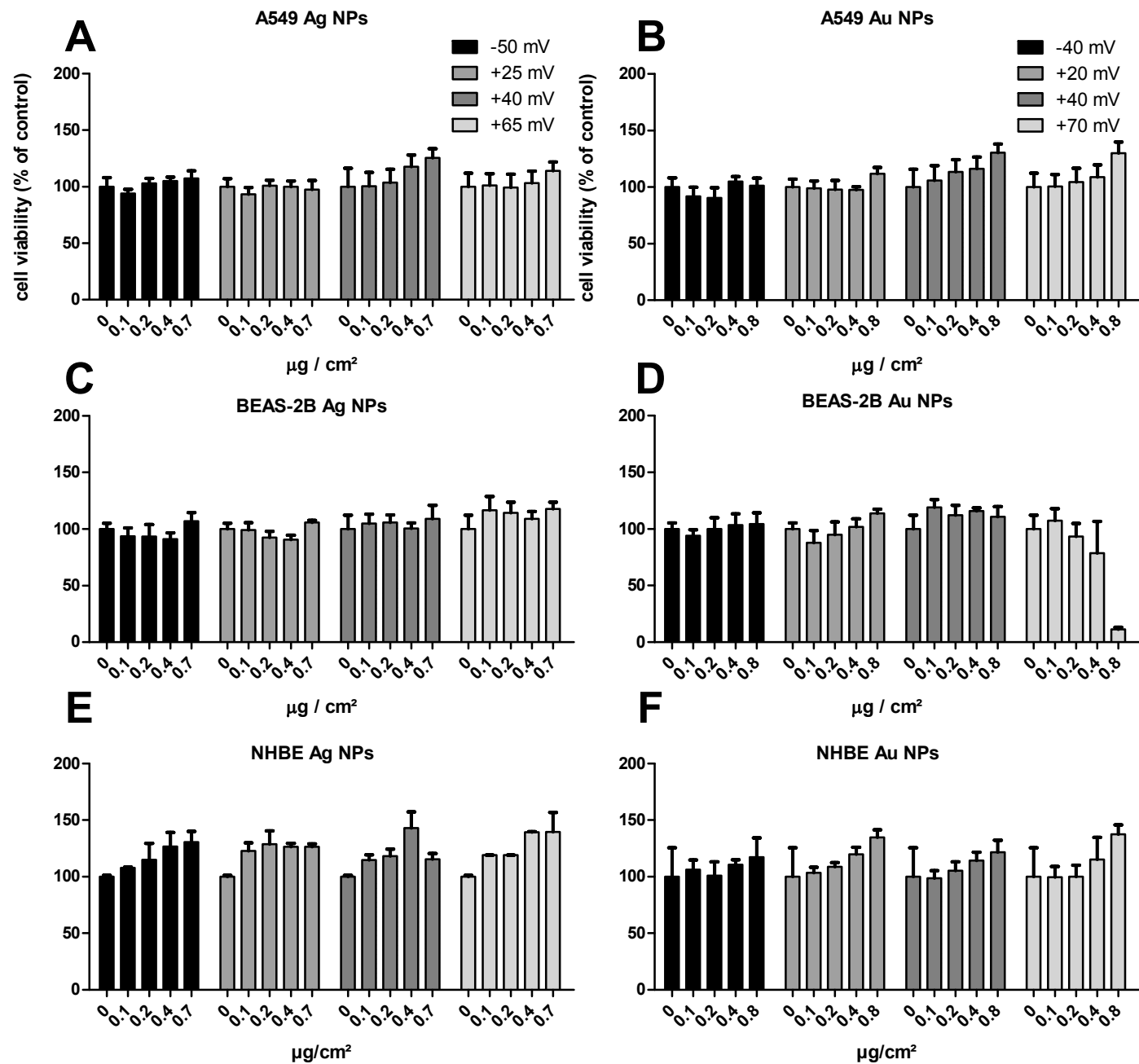

Supplement: Additional file 4: — Cell viability following a 4 h exposure to charged Ag and Au NPs of A549 cells (A, B; means ± SEM of n = 6), BEAS-2B cells (C, D; means ± SEM of n = 3) and NHBE cells (E, F; means ± SEM of n = 3). P-values * < 0.05, Cells treated with medium only were used as control (100%). [file 12951_2014_62_MOESM4_ESM.pdf]

**A549 Ag NPs**

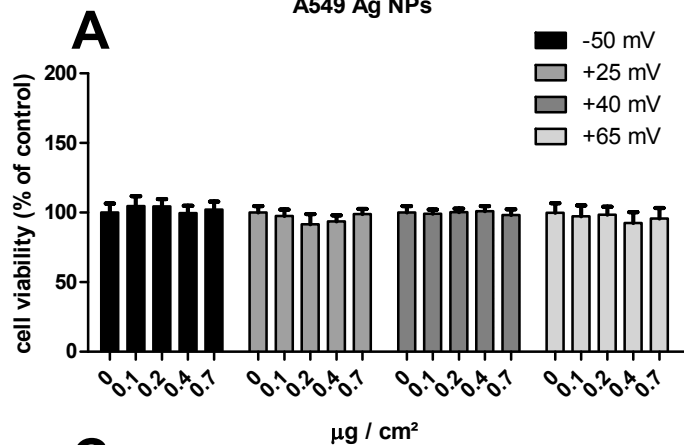

**A549 Au NPs**

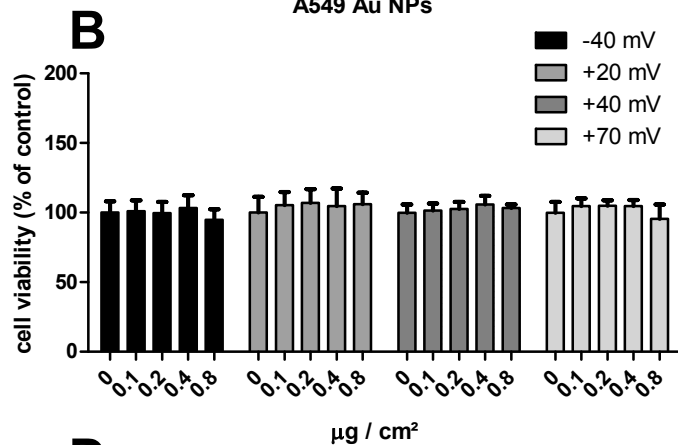

**BEAS-2B Ag NPs**

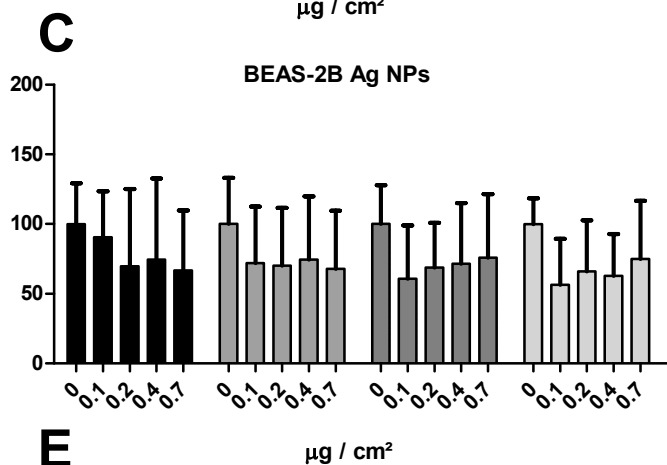

**BEAS-2B Au NPs**

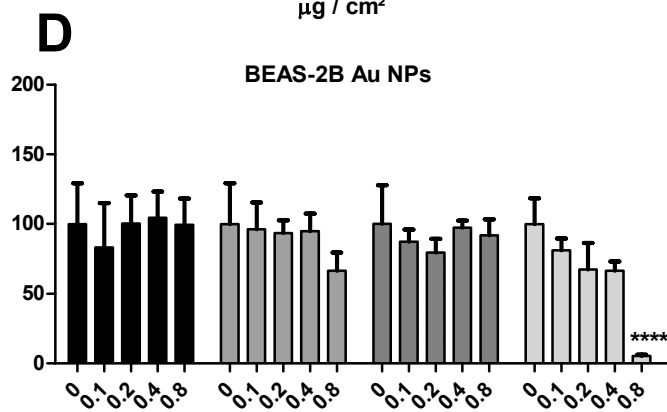

**NHBE Ag NPs**

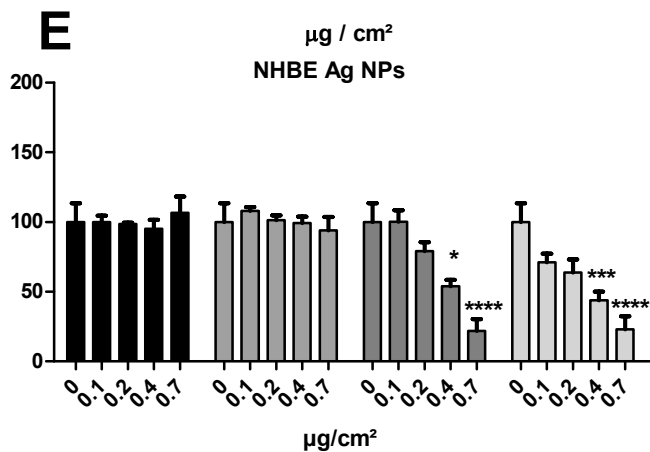

**NHBE Au NPs**

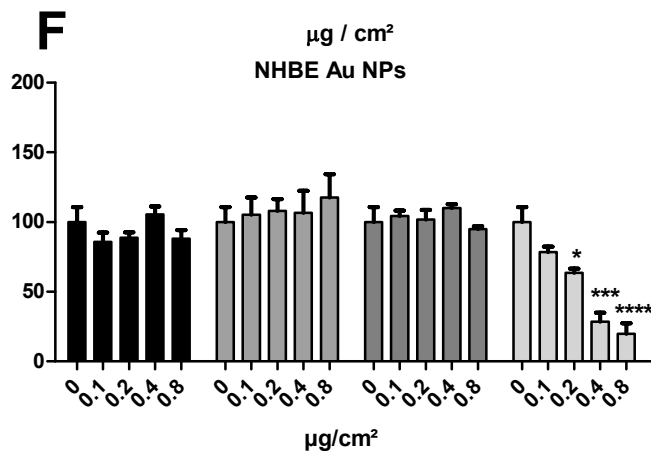

Supplement: Additional file 5: — Cell viability following a 48 h exposure to charged Ag and Au NPs of A549 cells (A, B; means ± SEM of n = 6), BEAS-2B cells (C, D; means ± SEM of n = 3) and NHBE cells (E, F; means ± SEM of n = 3). P-values * < 0.05, Cells treated with medium only were used as control (100%). [file 12951_2014_62_MOESM5_ESM.pdf]

**A****Ag NPs (1:40 dilution)**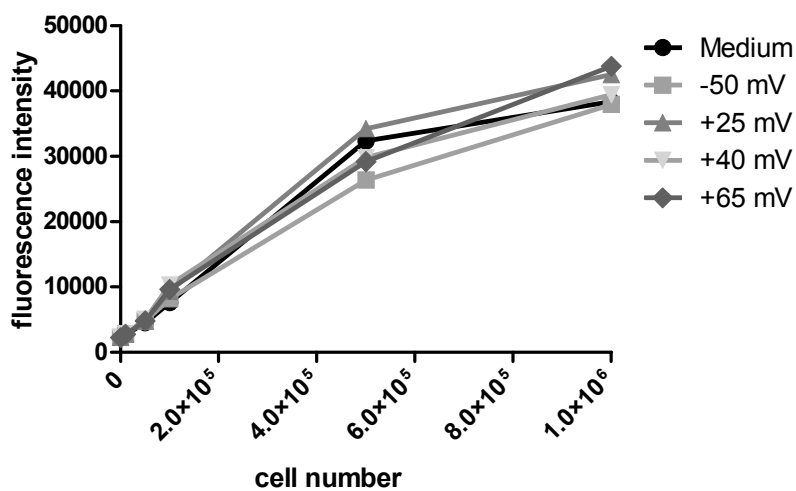**B****Ag NPs (1:10 dilution)**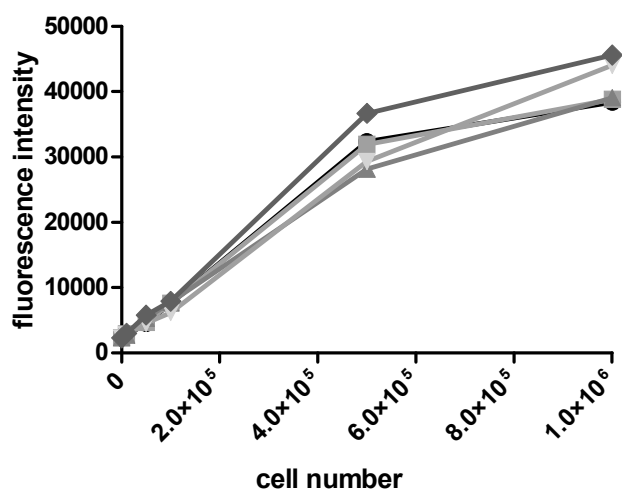**C****Au NPs (1:40 dilution)**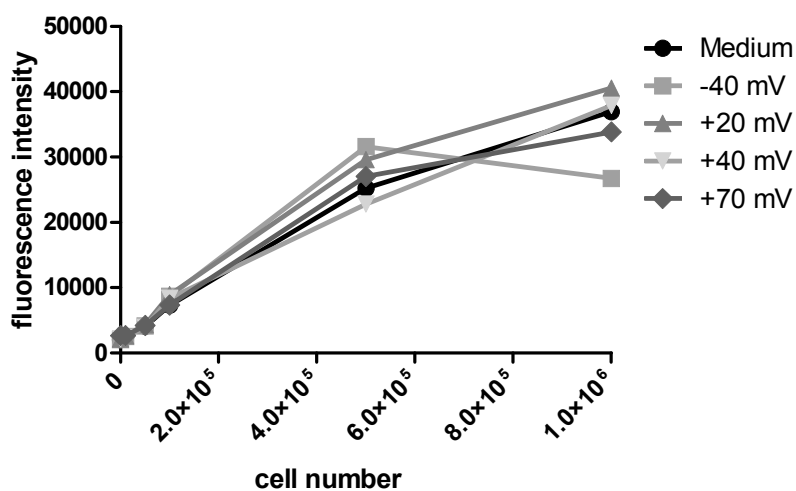**D****Au NPs (1:10 dilution)**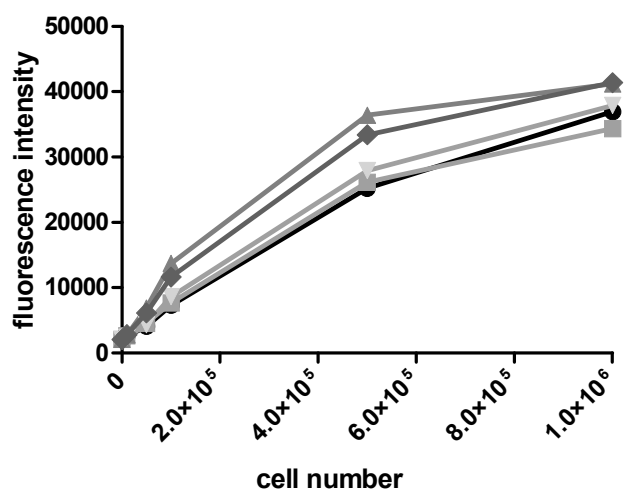

Supplement: Additional file 6: — Assessment of interference of Ag NPs (A, B) and Au (C, D) with the CTB assay. [file 12951_2014_62_MOESM6_ESM.pdf]

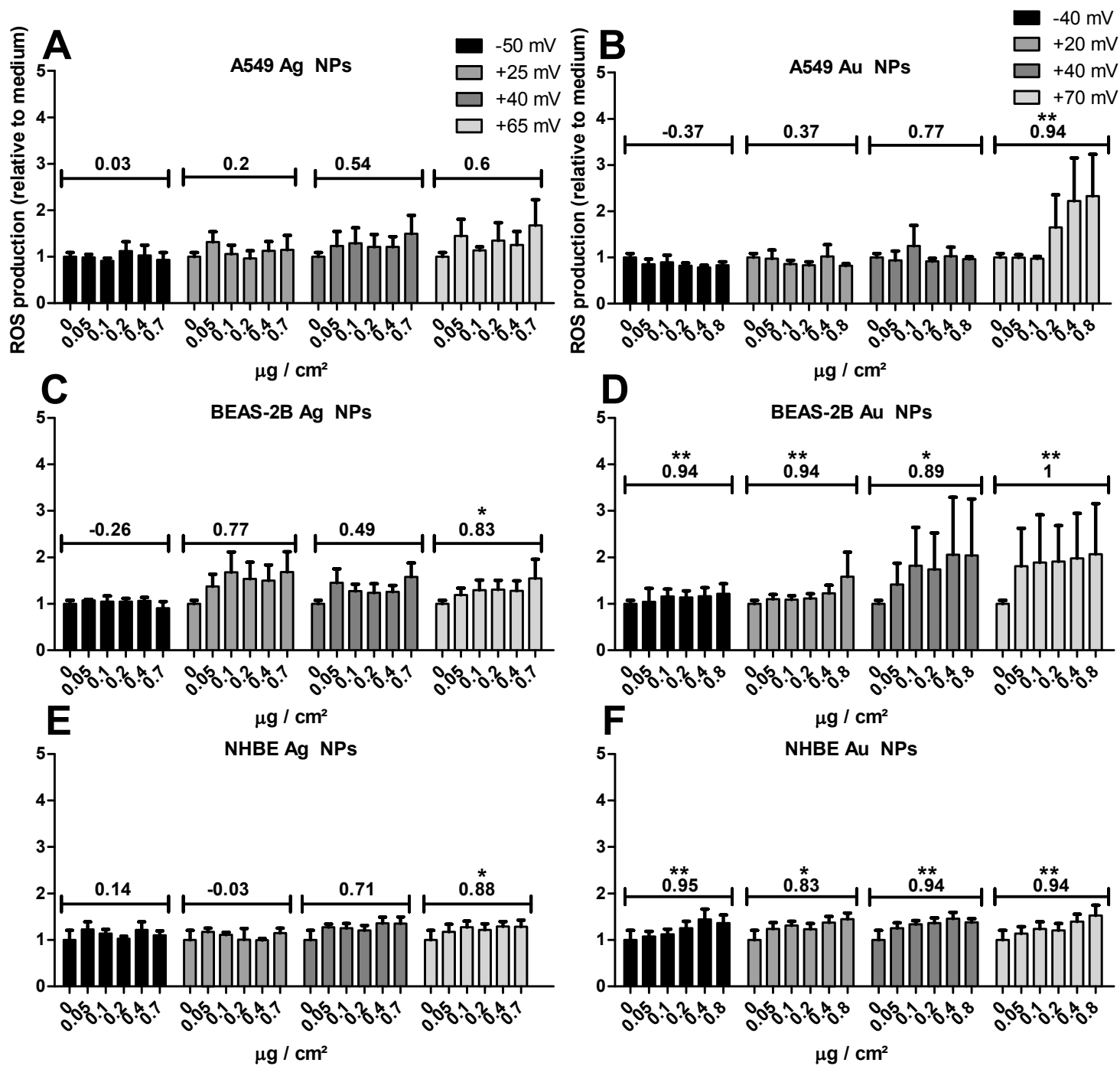

Supplement: Additional file 7: — ROS production measured using the DCFH-DA assay following a 1 h exposure of A549 cells (A, B, means ± SEM of n = 3), BEAS-2B cells (C, D, means ± SEM of n = 3) and NHBE cells (E, F means ± SEM of n = 4) to functionalized Ag and Au NPs. Spearman’s rank coefficients were calculated for each NP to assess possible charge dependent increases in ROS production. In addition, the coefficients of the highest concentrations of each NP surface charge were calculated to determine if ROS production was charge dependent. P-values * < 0.05, ** < 0.01. [file 12951_2014_62_MOESM7_ESM.pdf]

## Solvents

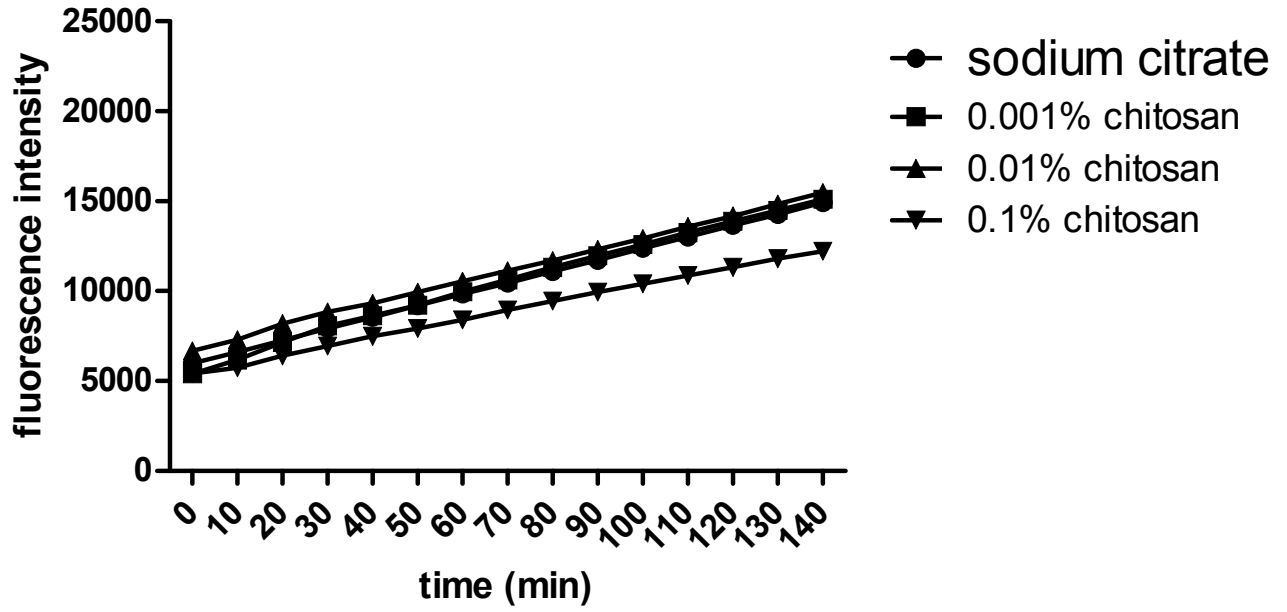

Supplement: Additional file 8: — ROS production of the NPs solvents in a cell-free system. Fluorescence was measured at 530 nm (excitation 485 nm) every 10 minutes after an initial incubation period of 15 minutes. Means ± SEM of n = 3 are shown. [file 12951_2014_62_MOESM8_ESM.pdf]
