# Supplementary material for: The oxidative potential of differently charged silver and gold nanoparticles on three human lung epithelial cell types
Source: J Nanobiotechnology. 2015 Jan 16;13:1. doi: 10.1186/s12951-014-0062-4 (PMC4304186; doi:10.1186/s12951-014-0062-4)
Supplement: Additional file 9: — Surface charge (mV) of NPs as synthesised and after a 4 hour incubation in different cell culture media. [file 12951_2014_62_MOESM9_ESM.pdf]

Sample	As synth	In A549 medium	In BEAS-2B medium	In NHBE medium
Ag-SC	-50	-30	-23	-35
Ag-CHIT-L	+25	-15	+21	-2
Ag-CHIT-M	+40	-17	+23	-5
Ag-CHIT-H	+65	-6	+30	+2
Au-SC	-40	-36	-25	-36
Au-CHIT-L	+20	-11	+19	-5
Au-CHIT-M	+40	-13	+16	-7
Au-CHIT-H	+70	-10	+21	-1
